# Current Approaches in Cosmeceuticals: Peptides, Biotics and Marine Biopolymers

**DOI:** 10.3390/polym17060798

**Published:** 2025-03-18

**Authors:** Ulya Badilli, Ozge Inal

**Affiliations:** Department of Pharmaceutical Technology, Faculty of Pharmacy, Ankara University, 06560 Ankara, Turkey; unuman@pharmacy.ankara.edu.tr

**Keywords:** cosmetic product, natural compounds, bioactive agents, peptides, biotics, marine ingredients, biopolymers

## Abstract

Today’s consumer perception and expectations of personal care have gone beyond merely cleansing, moisturizing, and makeup products, focusing more on the reduction or elimination of signs of aging. Cosmeceuticals, developed to create a more youthful appearance, commonly contain substances with therapeutic and physiological effects. The development of cosmeceutical products containing peptides, biotic ingredients, and marine-based compounds has become a highly popular strategy to enhance anti-aging effects and better address consumer demands. Peptides are frequently used in anti-aging products due to their effects on enhancing fibroblast proliferation and collagen synthesis, contributing to the skin’s barrier function, and reducing skin pigmentation. Meanwhile, biotic components are extensively evaluated for their potential to improve barrier function by maintaining the balance of the skin microflora. On the other hand, the increasing interest of cosmetic consumers in natural and eco-friendly products, along with the rich biodiversity in the oceans and seas, has made marine-derived substances highly significant for the cosmetic industry. Marine polysaccharides are particularly valuable as biopolymers, offering useful properties for gel formation in cosmetic formulations. This review discusses scientific studies and commercially available products using peptides, biotic and marine-based compounds in cosmetic formulations, their cosmetic and cosmeceutical benefits, and the challenges in the formulation design of these products.

## 1. Introduction

Cosmetics are defined by the Federal Food, Drug, and Cosmetic Act (FD&C Act) as “articles intended to be rubbed, poured, sprinkled, or sprayed on, introduced into, or otherwise applied to the human body… for cleansing, beautifying, promoting attractiveness, or altering the appearance”. The FD&C Act also defines a cosmetic product as “a preparation of cosmetic ingredients with a qualitatively and quantitatively set composition for use in a finished product”. According to the EU Cosmetic Products Regulation, a cosmetic is “any substance or mixture intended to be placed in contact with the external parts of the human body (epidermis, hair system, nails, lips, and external genital organs) or with the teeth and the mucous membranes of the oral cavity, with the exclusive or primary purpose of cleaning, perfuming, changing appearance, protecting, keeping in good condition, or correcting body odors”. As can be clearly understood from these definitions, cosmetics are an important part of personal self-care and serve the purposes of cleansing, moisturizing, enhancing beauty and attractiveness, and providing pleasant scents.

However, today’s consumer perception and expectations of personal care have gone beyond merely cleansing, moisturizing, and makeup products, focusing more on the reduction or elimination of signs of aging. Cosmeceuticals, which occupy a position between cosmetics and pharmaceuticals, are products designed to improve the appearance, texture, and function of the skin through physiological pathways. Although there is no official definition of “cosmeceuticals” in regulations, they can be defined as products that have restorative effects on the skin. Cosmeceuticals, developed to create a more youthful appearance by reducing signs of aging such as hyperpigmentation, wrinkles, dryness, and loss of elasticity, have become the fastest-growing segment of the personal care industry [1,2]. These products commonly contain substances with therapeutic and physiological effects, such as antioxidants, depigmentation agents, vitamins, sunscreens, and hydroxy acids. The usage of peptides, biotic ingredients, and marine-derived biopolymers has become a considerably novel approach in this area. This review comprehensively discusses cosmeceuticals containing peptides, biotics, and marine ingredients, evaluating both their benefits and challenges encountered in their production.

## 2. Peptides in Cosmeceuticals

Peptides were introduced to the cosmeceutical industry in 1973 when Pickart identified the synthetic signal peptide GHK which stimulates collagen synthesis and functions as a carrier peptide when complexed with copper ions. Since then, several peptides with potential cosmeceutical applications have been developed to meet the growing demands in the cosmetic market [3]. Novel cosmeceuticals including peptides or proteins that have biological functions provide substantial effects on skin beauty and improved skin appearance. Cosmeceutical peptides offer numerous anti-aging benefits, such as promoting collagen and elastin synthesis, boosting fibroblast proliferation, increasing skin hydration and barrier function, and reducing skin pigmentation, which has been verified by in vitro and in vivo studies [4,5,6,7]. These peptides, which can be derived from microorganisms, plants, marine sources, and animals or can be obtained synthetically, are classified based on their functionalities as signal, carrier, neurotransmitter-inhibitory, and enzyme-inhibitory peptides [7].

### 2.1. Signal Peptides

The name of signal peptides originates in the ability to mimick the signal occurring in the synthesis of extracellular matrix (ECM) proteins. These peptides released by the ECM that promote collagen production are also called matricins [3]. Signal peptides show anti-aging activity by stimulating skin fibroblasts, resulting in increased production of collagen, elastin, fibronectin, and glycosaminoglycan. They function as growth factors by activating protein kinase C, a key enzyme involved in regulating cell growth and migration [7]. The most commonly investigated signal peptides in cosmeceuticals are summarized below.

#### 2.1.1. Palmitoyl Pentapeptide-4 (Matrixyl^®^)

One of the first well-known signal peptides, KTTKS (Lys-Thr-Thr-Lys-Ser), is found in pro-collagen type I and stimulates the synthesis of the main components of the extracellular matrix (ECM) in the dermis [8]. However, due to its hydrophilic nature, KTTKS cannot easily penetrate the stratum corneum. Therefore, a palmitoyl derivative has been synthesized to increase lipophilicity and stability, as well as to provide an effective skin delivery [9]. Palmitoyl pentapeptide-4 (Pal-KTTKS) (Matrixyl^®^) (Sederma, Croda Int. Group, Snaith, UK) is frequently used as an anti-aging agent since it stimulates the production of collagen (especially types I and III), elastin, fibronectin, and glycosaminoglycans [10,11]. In a clinical study conducted over 12 weeks with 93 Caucasian female participants (aged 35–55), Pal-KTTKS demonstrated a significant reduction in wrinkles compared to the placebo control, as evidenced both quantitatively and through expert-rated visual analysis [12]. Osborne et al. (2005) also conducted a clinical study in which a daily facial moisturizer containing palmitoyl pentapeptide (Pal-KTTKS) was applied to 60 women aged 35 to 65 for 8 weeks. The results showed that the Pal-KTTKS-containing product led to a significant reduction in the appearance of rough texture and fine wrinkles in the skin at the 4th and 8th weeks [13].

#### 2.1.2. Palmitoyl Tripeptide-1 (Biopeptide CL^TM^)

Palmitoyl tripeptide-1 is a signal peptide synthesized by conjugating tripeptide-1 with palmitic acid. It acts as a carrier peptide when complexed with copper. It targets TGF-β and stimulates the production of ECM proteins by dermal fibroblasts, thereby reducing wrinkles. There are two products on the market containing palmitoyl tripeptide-1. The first is Biopeptide CL™ (Sederma, Croda Int. Group), which contains only palmitoyl tripeptide-1, and the second is Matrixyl™ 3000 (Sederma, Croda Int. Group), a combination of palmitoyl tripeptide-1 and palmitoyl tetrapeptide-7. Biopeptide CL™ has been reported to exhibit activity comparable to retinoids but without causing skin irritation [14]. A recent study evaluated the anti-wrinkle effectiveness of a peptide combination that contains palmitoyl tripeptide-1 and palmitoyl tetrapeptide-7. The study, conducted on 32 volunteers with an average age of 28.5 years, demonstrated a significant reduction in the depth, volume, and number of wrinkles at the end of 28 days [15].

#### 2.1.3. Palmitoyl Hexapeptide-12 (Biopeptide EL^TM^)

Palmitoyl hexapeptide-12 (Sederma, Croda Int. Group) (Pal-Val-Gly-Val-Ala-Pro-Gly) is an elastin fragment that stimulates collagen and elastin synthesis, providing an improved skin elasticity and firmness effect [16,17]. It is believed to function by reducing the production of interleukin-6 (IL-6) in keratinocytes and fibroblasts [11].

#### 2.1.4. Palmitoyl Tripeptide-5 (Syn^®^-Coll)

Palmitoyl tripeptide-5 (Syn^®^-Coll) (DSM, Basel, Switzerland) mimics the thrombospondin I (TSP-I) tripeptide sequence and can stimulate matrix protein production by upregulating transforming growth factor beta (TGF-β) [18,19]. It effectively supports collagen synthesis and improves skin elasticity and texture. Chen et al. (2025), developed supramolecular collagen nanoparticles composed of a combination of lactoferrin, recombinant human collagen, and palmitoyl tripeptide-5. In this structure, lactoferrin and recombinant human collagen were used as carriers to encapsulate palmitoyl tripeptide-5. These collagen nanoparticles showed significant moisturizing, firming, and whitening effects, and reduced the length of under-eye bags and nasolabial folds by 10.22% and 21.57%, respectively [20]. In another study, nanoliposome formulations combining palmitoyl tripeptide-5 (Syn^®^-Coll), Argireline^®^, and carnosine were developed to assess their effectiveness against skin aging. The results demonstrated that the combination of these peptides produced a synergistic effect, enhancing collagen production, inhibiting neurotransmitter release, and providing antioxidation. As a result, there was a noticeable reduction in wrinkle volume and improvement in skin elasticity [16].

#### 2.1.5. Tripeptide-10 Citrulline (Decorinyl^®^)

Tripeptide-10 Citrulline (Decorinyl^®^) (Lipotec, Barcelona, Spain) is a bioactive peptide that mimics the sequences of decorin, a natural leucin-rich proteoglycan of the dermal matrix and binds specifically to collagen fibrils. It has been shown to promote skin elasticity by regulating collagen fibrillogenesis and increasing collagen fibril stability [21,22]. As skin ages, the loss of functional decorin leads to disrupted collagen fibers and a decrease in the skin’s tensile strength. Tripeptide-10 Citrulline is an innovative cosmetic ingredient that ensures uniformity in collagen fibril diameter and enhances skin elasticity by improving the cohesion of collagen fibers [23].

### 2.2. Carrier Peptides

Carrier peptides are responsible for transporting trace elements such as copper and manganese into cells, which are crucial for maintaining cellular activities and several enzymatic processes. Many enzymes operate dependently on copper. Superoxide dismutase, a significant antioxidant that counters free radicals contributing to skin aging by damaging collagen, requires copper as a cofactor. Lysyl oxidase, which plays a crucial role in collagen and elastin production, is also a copper-dependent enzyme. Copper is also the cofactor for cytochrome-c oxidase and tyrosinase enzymes, which are important for skin care. The tripeptide glycyl-L-histidyl-L-lysine (GHK), which is a typical example of these peptides, spontaneously forms a complex with copper, facilitating the uptake of this element by cells. The resulting GHK-copper complex stimulates collagen synthesis by fibroblasts and increases levels of MMP-2 and tissue inhibitors of metalloproteinases (TIMPs) 1 and 2. Consequently, it promotes the remodeling of dermal tissue. On the other hand, the tripeptide GHK alone has been shown to enhance collagen production. This tripeptide, found in the alpha II chain of human collagen, is released from the collagen helix in response to injury or inflammation and stimulates new collagen synthesis. While GHK alone is effective as a signal peptide, its use as a copper peptide complex is considered to be more effective [6,24].

#### 2.2.1. Copper Tripeptide-1 (Cu-GHK)

Copper Tripeptide-1 stimulates collagen, elastin, and glycosaminoglycan synthesis in the skin, reduces wrinkles and hyperpigmentation, and accelerates wound healing. Administration of Cu-GHK has been shown to increase extracellular matrix deposition in a rat wound model. While the most-studied effects of Cu-GHK are skin rejuvenation and wound healing, it also stimulates hair growth and is used to improve the success of hair transplantation procedures [25,26]. Copper Tripeptide-1 has also been shown to exhibit significant antioxidant effects in in vitro and in vivo studies. It may contribute to the antioxidant defense mechanism of the skin by promoting superoxide dismutase activity, neutralizing toxic products of lipid peroxidation, and modulating the expression of multiple antioxidant-related genes [27].

In a study performed by Badenhorst et al. (2016), human dermal fibroblasts were incubated with Copper Tripeptide-1 at concentrations of 0.01, 1, and 100 nM. mRNA gene expression levels of metalloproteinases (MMP1, MMP2) and tissue inhibitors of metalloproteinases (TIMP1, TIMP2) in treated and control dermal fibroblasts were measured. It was observed that Copper Tripeptide-1 significantly upregulated MMP1 and MMP2 gene expression at the lowest concentration while increasing TIMP1 expression at all concentrations. All concentrations of copper peptide resulted in enhanced production of collagen and elastin. The elevated mRNA expression ratio of TIMPs to MMPs correlated with collagen and elastin synthesis stimulation [28]. In another study, it has been shown that the combination of Cu-GHK and hyaluronic acid supports the production of collagen I, IV, and VII, creating a significant synergistic effect, particularly for collagen IV synthesis. It has been noted that when an appropriate hyaluronic acid molecular weight and Cu-GHK/hyaluronic acid ratio are used, a significant increase in collagen IV synthesis can be achieved [29]. Recent research on Cu-GHK-loaded liposomes demonstrated a 48.90% inhibition of elastase activity. Consequently, it reduced elastin degeneration and helped maintain the structural integrity of the skin [30]. Another study investigating the effects of a peptide mixture containing Copper Tripeptide-1 (0.05%) on hair cycle regulation by hair follicle culture concluded that this peptide mixture was beneficial in promoting hair growth by stimulating the anagen phase and increasing cell proliferation [31].

#### 2.2.2. Manganese Tripeptide-1 (Mn-GHK)

Manganese, an essential trace element that plays a role in various metabolic reactions and antioxidant protection, has been shown to be beneficial in the prevention of photoaging, as have copper peptide complexes. A serum formulation containing Manganese Tripeptide-1, used twice daily for 12 weeks, has been shown to significantly improve the appearance of hyperpigmentation associated with photoaging in the facial area [32].

### 2.3. Neurotransmitter Inhibitory Peptides

The wrinkles and fine lines, which are the most common signs of aging, can be prevented or reduced using neurotransmitter inhibitor peptides. Acetylcholine-containing vesicles must be released from the neuromuscular junction and interact with SNARE complexes for muscle contraction to occur. This process is modulated by SNAP-25, a membrane protein that regulates the binding and vesicle fusion of SNARE complexes. Neurotransmitter inhibitory peptides, which are also referred to as “botox-like peptides”, exhibit specific neurosuppressive properties. They show structural similarities to the SNAP-25 protein and compete for the binding sites of SNARE complexes, inhibiting the release of acetylcholine at the neuromuscular junction and preventing muscle contraction [4].

#### 2.3.1. Acetyl Hexapeptide-3 (Argireline^®^)

One of the most popular commercial neurotransmitter inhibitory peptides is acetyl hexapeptide-3, which has a sequence of Acetyl-Glu-Glu-Met-Gln-Arg-ArgNH_2_. This peptide derived from SNAP-25 is marketed under the name Argireline^®^ (Lipotec S.A., Barcelona, Spain) as a biocompatible alternative for reducing facial wrinkles. Argireline^®^ shows a similar effect to botulinum toxin, that is, competing for binding to the SNARE complexes, inhibiting the release of acetylcholine and, as a result, muscle contraction [4,7,33]. It is reported to be 4000 times less toxic than botulinum toxin and significantly improves the mechanical properties of the skin without the toxin-related side effects [34,35,36].

The effectiveness of Argireline^®^ in improving skin health and reducing or preventing signs of aging has been demonstrated through clinical studies [37]. Results of a study conducted in China showed that applying Argireline^®^ to the eye contour for 4 weeks was effective in reducing the severity of moderate to severe periorbital wrinkles [38]. In another study, the effectiveness of Argireline^®^ on wrinkles was evaluated in vivo using an emulsion formulation for normal to dry skin and a gel formulation for oily skin. A significant reduction in the depth and width of wrinkles, as well as an increase in the moisture level of the skin, was confirmed in each volunteer [39]. The usage of Argireline^®^ in combination with other peptides was also evaluated. For example, it was reported that the combination of acetyl hexapeptide-3 and tripeptide-10 citrulline, which act through different mechanisms, demonstrated synergistic clinical efficacy on the skin [40].

#### 2.3.2. Tripeptide-3 (Syn-Ake^®^)

Syn-Ake^®^ (Pentapharm, Basel, Switzerland), a synthetic tripeptide (β-Ala-Pro-Dab-NHBn-2-Acetate) that mimics the effect of Waglerin-1, a 22-amino acid peptide obtained from the venom of *Tropidolaemus wagleri*, is a reversible antagonist of the muscle acetylcholine receptor. It is marketed in cream and serum formulations for wrinkle reduction by inhibiting muscle contractions [3,11,33,41]. Tripeptide-3, which has a Botox-like effect, has been reported to be an effective peptide in controlling excessive sebum secretion [42].

#### 2.3.3. Pentapeptide-3 (Vialox^®^)

Pentapeptide-3 (Vialox^®^) (Gly-Pro-Arg-Pro-Ala-NH_2_), an acetylcholine receptor antagonist derived from snake venom, exerts its effect by blocking nerves at the postsynaptic membrane and leading to muscle relaxation [3,11].

#### 2.3.4. Pentapeptide-18 (Leuphasyl^®^)

Leuphasyl^®^ (Pentapeptide-18) (Lipotec S.A., Barcelona, Spain) mimics the mechanism of enkephalins, which are endogenous ligands that bind to the opioid receptors of the body. This peptide (Tyr-D-Ala-Gly-Phe-Leu) inhibits the release of acetylcholine by blocking the calcium channels in neurons, thereby reducing the depth of wrinkles [8].

In a study where emulsions containing Leuphasyl^®^ at three different concentrations were applied to 20 volunteers over a 2-month period, it was shown that Leuphasyl^®^ has proven efficacy at a minimum concentration of 2%. Although the efficacy of this molecule is lower than that of botulinum toxin injections, it offers the advantages of being free from side effects and preserving facial expression. It has also been suggested that a mixture of Leuphasyl^®^ and Argireline^®^ is ideal for creating a longer-lasting synergistic effect [43].

A night face cream formulation containing solid lipid nanoparticles (SLNs) loaded with Leuphasyl^®^ and retinol was recently developed by [44]. After 8 weeks of application to 20 female volunteers aged 30–65, the anti-aging efficacy was evaluated using non-invasive methods. The reduction in the number, length, and depth of wrinkles indicated that the developed product improved the general condition of the skin, leading to a significant improvement in skin elasticity, thus proving the anti-aging efficacy [44].

#### 2.3.5. Acetyl Octapeptide-3 (SNAP-8)

Acetyl octapeptide-3 (SNAP-8) (Lipotec S.A., Barcelona, Spain) is produced by adding a chain of alanine and aspartame to the structure of acetyl hexapeptide-3 (Argireline^®^). It inhibits the release of acetylcholine by competing with SNAP-25, thereby preventing the formation of wrinkles [14,45].

In a recent study, an anti-aging microneedle patch containing acetyl octapeptide-3 and a hyaluronic acid (HA) polymer backbone was produced and applied to the eye area of twenty-four healthy volunteers for 28 days. Significant improvement in eye wrinkles, reduction in TEWL, and enhanced skin elasticity were observed, with no side effects reported [46].

### 2.4. Enzyme Inhibitory Peptides

Enzyme-inhibitory peptides can directly or indirectly inhibit enzymes, such as MMPs, that contribute to the aging of skin by breaking down collagen and other extracellular matrix (ECM) proteins. Soy oligopeptides, rice peptides, and silk fibroin peptides are enzyme-inhibitory peptides that are used in cosmeceuticals [7,14].

Rice peptides, which are obtained from rice-bran protein, inhibit the MMP activity and stimulate the expression of hyaluronan synthase 2 genes in human keratinocytes [7]. Chen et al. (2021a) demonstrated that rice peptides effectively inhibit the activities of tyrosinase and hyaluronidase enzymes. The results of the study revealed that rice peptides have a high potential to be used in anti-aging cosmeceuticals, serving antioxidant, antihyaluronidase, and antityrosinase purposes [47]. The results of a clinical study conducted on 60 female subjects aged 20–30 years to evaluate the effects of cosmetic products containing rice peptides and ascorbyl tetraisopalmitate on skin photoaging showed that daily use of these formulations increased epidermal cell renewal and skin moisture while reducing hyperpigmentation. Rice peptides were stated as compounds that promote cell renewal as well as having antioxidant properties [48].

Silk fibroin peptides are obtained from the silkworm and generally consist of glycine, alanine, serine, and tyrosine amino acids [14]. Sericin, the water-soluble component of silk, contains polypeptides with a molecular weight of 60–130 kDa and a high proportion of hydroxyl, carboxyl, and amino groups. Silk fibroin, the main component of silk, consists of polypeptides with a molecular weight of 25–350 kDa, and its amino acid composition is primarily made up of glycine, alanine, serine, and tyrosine. The diversity in the amino acid content of sericin is responsible for its free radical scavenging properties, antityrosinase activity, and stimulation of cell proliferation. It has also been demonstrated that sericin, which counteracts skin aging by increasing the levels of collagen types I and III, has high biocompatibility [49]. On the other hand, silk fibroin is a biocompatible natural protein hydrolysate that exhibits excellent water binding and absorption capacity [50].

Soy peptides are produced by enzymatic digestion or fermentation of soybean proteins [51]. These peptides consist of three to six amino acids and are often used as anti-aging agents, hair growth promoters, and skin moisturizers [7]. Soy peptides, rich in aspartic acid and glutamic acid, have been reported to increase collagen type I gene expression and promote collagen synthesis in human dermal fibroblasts [52,53]. In another study, soybean oligopeptides have been shown to significantly reduce the erythema index caused by UVB rays and prevent radiation-induced moisture loss. These results demonstrate the potential of soybean oligopeptides to protect human skin from UV radiation-induced damage [51]. In Table 1, some marketed cosmeceutical products containing peptide ingredients are summarized.

### 2.5. Challenges of Using Peptides in Cosmeceuticals

The development of advanced peptide-based products offers numerous remarkable benefits to the cosmetic industry but also presents various challenges. One issue that must be considered is ensuring the preservation of both the structural stability and the bioactivity of peptides during the production and storage of the products. It is important to note that peptides can be significantly affected by various factors such as pH, temperature, and interactions with other active ingredients [7]. On the other hand, peptides, with relatively high molecular weight and hydrophilic properties, face significant challenges in penetrating the stratum corneum layer of the skin [55,56]. Various strategies are employed to enhance the anti-aging effects of peptides by facilitating their penetration–permeation into the skin. These approaches can be categorized into physical and chemical methods for penetration enhancement, chemical modifications of the peptide structure to impart lipophilic properties, and advanced formulation strategies [14,57,58]. Chemical enhancers are compounds that temporarily weaken the barrier properties of the stratum corneum. This occurs through their interaction with the lipids of the stratum corneum, promoting the penetration of peptides into the skin [14]. In a study in which hydrogel formulations containing the N-Pro-Val-Ala-Gly tetrapeptide, which has the potential to stimulate dermal fibroblast growth and promote wound healing, were developed, it was reported that the penetration of the tetrapeptide could be modified by the hydrogel formulated with lemon essential oil [59]. Chemical modification to enhance lipophilicity and thereby improve the skin penetration of peptides is commonly achieved by conjugating them with lipophilic moieties such as palmitic, stearic, lauric, and oleic acids [58,60].

It is also possible to enhance the penetration of peptides through the skin via metal complexation. Dissette et al. (2018) prepared different gel formulations containing carnosine dipeptide or its complex with magnesium ions. They demonstrated that after the application of the carnosine metal complex, the concentration of carnosine detected in the receptor phase increased by 1.5 times compared to free carnosine [61]. The results of the study conducted by Park et al. (2020) also demonstrated that the penetration of GHK-Cu through the skin was higher than that of GHK, indicating the effect of metal complexation on the skin permeability of peptides [62].

Physical penetration enhancement strategies include sonophoresis, iontophoresis, electroporation, and microneedle applications. One of the most popular strategies for physical penetration enhancement is the use of microneedles. Microneedle-based peptide delivery is being evaluated as a current approach for the efficient penetration of hydrophilic peptides, depending on the molecular weight, into the skin through the stratum corneum [63]. In a study evaluating dissolving microneedles as a minimally invasive transdermal application, a clinical evaluation was conducted on 30 healthy subjects using polypeptide-loaded dissolving microneedles to improve facial wrinkles for 84 days. Compared with a placebo, polypeptide-loaded microneedles showed significant improvements in wrinkles at the corners of the eyes, under the eyes, and in the nasolabial folds. Dissolving microneedles were also shown to possess high biosafety and skin compatibility [64]. The microneedle patch with acetyl octapeptide-3 loaded onto a hyaluronic acid (HA) polymer backbone, developed by Shin et al. (2024), was shown to be more effective than traditional formulations in increasing percutaneous absorption of the peptide and could represent a new strategy for preventing or reducing skin wrinkles and other signs of aging [46]. Another study, presenting the microneedle-based application evaluated the anti-wrinkle efficacy of a combination of ingredients, including a mixture of several peptides, antioxidants, and seaweed extracts, by employing hyaluronic acid-based microneedles on volunteers. This application was shown to improve skin appearance significantly. While a 25.8% reduction in fine wrinkles was observed, a 15.4% increase in skin moisture was achieved [65]. Li et al. (2015) also reported that the skin permeability of Copper Tripeptide-1 and similar peptides can be effectively and safely increased with pre-treatment of microneedles [66].

Drug delivery systems are also comprehensively evaluated for increasing the skin penetration and permeation of cosmeceutical peptides. It has been shown that the enhanced skin permeation of Copper Tripeptide-1 can be achieved by nanoemulsion formulations. The nanoemulsion formulation increased the permeability of copper peptide, resulting in a 21.89% release of the active substance after 8 h of application. The nanoemulsion, due to its small droplet size and the surfactants used in the formulation, interacts with the stratum corneum layer, reducing its barrier function and facilitating the penetration of copper peptide through the skin [67]. In a study, aiming to increase the penetration of hydrophilic Tripeptide-3 by encapsulation in microemulsions and nanoemulsions for reducing the sebum secretion on the face, nanoemulsions showed higher in vitro skin penetration and retention. A clinical study was also conducted on volunteers with oily facial skin, and an approximately 20% decrease in sebum production was observed after 28 days of nanoemulsion application [42]. The encapsulation of anti-aging peptides, including acetyl hexapeptide-8, palmitoyl pentapeptide-4, and palmitoyl tripeptide-1, in liposomes, combined with low-energy radiofrequency application, was evaluated to enhance skin penetration and improve anti-aging effectiveness. This strategy resulted in a permeation amount of peptides that was 3.65 times higher compared to free peptides. Clinical investigation results also demonstrated that the peptide-loaded liposome formulation significantly improved anti-wrinkle and skin-tightening effects [68]. GHK tripeptide-loaded poly(lactide-co-glycolide) (PLGA) nanoparticles were prepared to enhance the stability of the peptide, improve its skin penetration and efficacy, and provide controlled release in the targeted region. In vitro cell culture studies of both GHK tripeptide and GHK-loaded PLGA nanoparticles were conducted. Cytotoxicity results on L929 cells indicated that GHK-loaded PLGA nanoparticles maintained over 80% cell viability [69]. In another study, solid lipid nanoparticles (SLNs) incorporating the P7 heptapeptide, which corresponds to the Keap1 binding motif for Nrf2 interaction, were produced using the hot high-pressure homogenization method. Dermatological results demonstrated that the SLNs effectively delivered the active peptide into skin cells and induced Nrf2 activation in dermal tissues [70].

## 3. Cosmeceuticals with Biotic Ingredients

Micro-ecological skin care is a current concept that essentially refers to accelerating skin regeneration, enhancing the skin’s barrier function, and maintaining skin in good condition through biotic support [71].

The skin, the external organ of the body, hosts a wide variety of organisms, including bacteria, yeasts, viruses, fungi, archaea, and mites [72]. An individual’s skin microflora is formed by transfer from the vaginal flora during vaginal birth or from the environmental flora during cesarean section. This composition of skin microflora then gradually evolves and varies from individual to individual according to age, gender, ethnicity, genetic and environmental influences, geographical location, immune status, lifestyle habits, hygiene routine, and cosmetic usage. It develops differently across various body parts, such as the face, armpits, and back. The skin microflora is generally composed of commensal and transient microorganisms. Transient microorganisms include opportunistic pathogens, while commensals are mostly considered beneficial, although they can become virulent under the influence of endogenous or exogenous factors. The commensal microflora of the skin plays an important defense role against pathogens and is crucial for immunity. Disruption of the balance in the microflora can lead to significant skin diseases [73,74,75,76]. For instance, *Staphylococcus epidermidis* is one of the predominant species in the skin microbiota, which is beneficial to the host. However, when dysbiosis occurs, it may contribute to the development of atopic dermatitis [77]. The commensal microflora mainly consists of Gram-positive bacteria such as *Propionibacterium* (now known as *Cutibacterium)*, *Corynebacterium*, and *Staphylococcus*. Gram-negative *Acinetobacter* species are commensal bacteria with limited distribution on the skin. *Malassezia furfur* is a yeast found on most skin sites with high prevalence, representing 80% of fungi. These microorganisms are found in the superficial layers of the stratum corneum, while *Propionibacterium (Cutibacterium)*, *Staphylococcus*, and *Malassezia* species are also present in the sebaceous follicles. Viruses can also be found in low concentrations on the skin surface, such as β and γ human papillomaviruses, which are among the most prevalent viral components of the skin microbiota [75,76,77,78].

Recent studies suggest that certain probiotic strains may provide benefits, such as improving skin barrier function and preventing the development of skin diseases by maintaining the balance of the skin microflora. As a result, biotic components have become increasingly popular in skin care products in recent years [75].

### 3.1. Probiotics

The International Scientific Association of Probiotics and Prebiotics (ISAPP) defines probiotics as “live microorganisms that, when administrated in adequate amounts, confer a health benefit on the host” [79,80]. The most commonly used probiotics belong to the *Lactobacillus* and *Bifidobacterium* genera. Notable species include *Bifidobacterium infantis*, *Bifidobacterium animalis* subsp. *lactis*, *Bifidobacterium longum*, *Lactobacillus rhamnosus*, *Lactobacillus acidophilus*, *Lactobacillus plantarum*, *Lactobacillus casei*, and *Lactobacillus delbrueckii* subsp. *Bulgaricus*. Additionally, probiotics may also comprise other bacterial strains such as *Propionibacterium acidilactici*, *Lactococcus lactis*, *Leuconostoc mesenteroides*, *Bacillus subtilis*, *Enterococcus faecium*, *Streptococcus thermophilus*, and *Escherichia coli*, as well as yeasts like *Saccharomyces boulardii* [77].

Probiotics are considered non-pathogenic microorganisms, meaning that they are assumed not to cause any adverse effects on the host, regardless of the source from which they are derived. Species such as *Lactobacillus*, *Bifidobacterium*, and *Lactococcus* are recognized as GRAS (Generally Recognized As Safe) in fermented foods and beverages. On the other hand, the safety of probiotics may depend on factors such as the type of microorganism and the condition of the individual (e.g., age, immunosuppression therapy, or pregnancy) [79].

Probiotic microorganisms from the genera *Bifidobacterium*, *Lactobacillus*, *Lactococcus*, and *Bacillus* hold promise for cosmetic care of various skin types. *Bacillus* species can be utilized for the care of oily skin prone to acne formation, while *Bifidobacterium* species are considered “universal probiotics” and can be applied in cosmetic products for all skin types [81]. The skin whitening, moisturizing, anti-aging, and anti-wrinkle effects of probiotics such as *Lactobacillus*, *Bifidobacterium*, and *Streptococcus* have been widely studied [71]. *Lactobacillus* species have been reported to enhance skin moisture and elasticity while reducing the appearance of wrinkles [79]. On the other hand, in a study conducted by Notay et al. (2020), the efficacy of a topical product containing *Nitrosomonas eutropha* in reducing facial wrinkles was evaluated. The results indicated that the probiotic-based product significantly reduced both the depth of facial wrinkles and hyperpigmentation in the forehead area [82].

Probiotics can also regulate skin pH, improve the skin’s barrier function, and reduce photoaging by alleviating oxidative stress [83]. *Lactobacillus* and *Bifidobacterium* strains possess free radical scavenging properties, which are associated with the accelerated repair of UV-exposed keratinocytes. Therefore, these probiotics can be used in cosmetic skin care products to prevent photoaging [84]. Probiotic products must fulfill some critical criteria. First, the strain selected must be phenotypically and genetically characterized for its specific intended application. On the other hand, the product must maintain a viable concentration of microorganisms equivalent to that shown in clinical trials, and it must confer measurable benefits throughout its use. Finally, dosage and duration of use should be established based on data derived from human clinical studies [80].

### 3.2. Postbiotics

The ISAPP describes postbiotics as “a preparation of inanimate microorganisms and/or their components that confer a health benefit on the host” [79,85]. Postbiotics, which can also be defined as “soluble factors secreted by living bacteria or released by bacterial lysis”, refer to metabolic byproducts such as bacterial lysates, cell wall fragments, short-chain fatty acids, enzymes, vitamins, exopolysaccharides, bacteriocins, cell surface proteins, peptides, peptidoglycan-derived muropeptides, amino acids, teichoic acid, and organic acids (e.g., lactic acid) produced by a probiotic organism during their lifespan [73,79,85,86,87].

Postbiotics are primarily derived from microorganisms of the *Lactobacillus* and *Bacillus* genera, as well as yeast species, particularly *Saccharomyces cerevisiae*. However, other probiotic bacteria such as *Bifidobacterium*, *Lactococcus*, *Enterococcus*, and *Leuconostoc* species can also be used as safe sources of postbiotics [79,88]. Postbiotics are produced during the feeding of probiotics with prebiotics, or during processes such as pasteurization, fermentation, or metabolic activities. These components can also be obtained in laboratory settings using methods such as UV or ionizing radiation, high temperature, high pressure, sonication, or inactivation with formalin [79,85]. Postbiotics, exhibiting a range of biological properties from anti-inflammatory to antioxidant effects, offer notable advantages over probiotics, including simpler preparation, large-scale production, higher purity, and extended shelf life [85,89].

The results of a study investigating a cream containing *Streptococcus thermophilus* lysate revealed an enhancement in both ceramide levels and moisture content in the stratum corneum [90]. The topical application of the postbiotic *Epidermidibacterium keratini* ferment filtrate on the faces of healthy female volunteers significantly improved skin barrier function, as well as skin elasticity and dermal density [91]. In another study, Lactococcus Ferment Lysate and Bifida Ferment Lysate postbiotics were compared with conventional synthetic chemicals in terms of their potential to protect against free radicals and UV rays. It was shown that Lactococcus Ferment Lysate and Bifida Ferment Lysate inhibited 54.2% and 71.0% of the DPPH (2,2-diphenyl-1-picrylhydrazyl) radical, respectively. The emulsion formulation (SPF 4.75) containing 8% Lactococcus Ferment Lysate and the reference emulsion (SPF 4.45) containing 8% of the chemical UVB filter, homomenthyl salicylate, were confirmed to have comparable UVB ray absorption capabilities [84].

### 3.3. Prebiotics

The experts of ISAPP recommended a new definition of prebiotics in 2016: “a substrate that is selectively utilized by host microorganisms, conferring a health benefit” [86,92]. In skin care, prebiotics represent fermentation metabolites that have a positive impact on maintaining a healthy skin microbiome, serving as nutrients for the “beneficial bacteria” on the skin [89]. In cosmeceutical products, prebiotics such as fructooligosaccharides, mannooligosaccharides, β-glucans, and lactulose are widely used. These prebiotics serve as food for probiotic bacteria and fungi [88].

A study conducted on healthy adults who applied a galacto-oligosaccharide-containing serum for 8 weeks revealed that the presence of prebiotics can positively alter the composition and diversity of skin microbiota. The application of the galacto-oligosaccharide-containing formulation effectively improved various properties of the skin and increased the population of beneficial microorganisms on the skin [93]. In another study, it was shown that galacto-oligosaccharides have high potential as skin prebiotics when applied at appropriate concentrations. Specifically, galacto-oligosaccharides at a concentration of 5% (*w*/*v*) stimulated the growth of beneficial *S. epidermidis* and inhibited the growth of *S. aureus*. These findings highlight the potential of galacto-oligosaccharides for positive regulation of the skin microbiota [94]. The triple biotic technology developed by Li et al. (2021) consists of a combination of inulin, 2-butyloctanol, and a biomimetic postbiotic blend. This triple biotic technology has been formulated into a body wash gel and a body lotion. In vitro and ex vivo studies have shown that the triple biotic technology provides an enhanced skin barrier by inhibiting the growth of undesirable bacteria and supporting the growth of desirable bacteria [95].

In Table 2, some examples of commercial cosmeceutical products with biotic ingredients are listed [74,96].

### 3.4. Challenges of Using Probiotics in Cosmeceuticals

Improving skin condition and enhancing skin appearance through balancing the skin microbiota is a strategy that has gained significant popularity and widespread consideration in recent years. However, claims of “balancing the microbiota” in biotic-containing cosmeceuticals should be approached with caution. Since there is no single type of healthy skin microbiota, the requirements for ‘balancing’ the microbiota of an individual are not straightforward. A product claiming to “balance” the microbiota should be developed based on extensive clinical studies involving hundreds of healthy volunteers. Considering that the likelihood of such studies being conducted for most products claiming to balance the microbiota is very low, the use of this claim is not appropriate, since it may have a risk of creating a false perception among consumers [97].

In order for a product to be labeled as “containing probiotics”, it must contain live probiotics throughout its shelf life. However, studies have revealed that an increasing number of products on the market, which claim to contain probiotics, are actually composed mainly of postbiotics and prebiotics instead of living bacteria, due to the difficulties in preserving the viability of probiotics. This situation leads to confusion and misinterpretation among consumers [98]. Maintaining the viability of probiotics in cosmetics from production to consumer use presents a significant challenge for the cosmetic industry [99]. The selection of a suitable carrier may provide a useful approach to overcome this issue. The effects of carriers such as emulgels, organogels, and hydrogels on the viability of probiotics have been evaluated by several researchers. Sharma et al. (2021) developed a topical emulgel formulation containing *Bacillus coagulans* to achieve the preservation of probiotic viability. They reported that they successfully formulated an emulgel that preserves the viability of probiotics, ensures germination, and exhibits wound-healing properties [100]. Letocha et al. (2024) investigated the potential of using organogels as carriers for protecting probiotics against stressful conditions and preserving their viability. They concluded that the formulation of encapsulated *Lactobacillus casei* strains in an organogel is a promising approach for maintaining probiotic viability [101].

Freeze-drying (lyophilization) is another commonly used method to preserve the viability of probiotic strains. The most frequently used cryoprotectants in this method include trehalose, glucose, sucrose, lactose, glycerol, polyethylene glycol, and skim milk. However, these cryoprotectants may not always be compatible with the physicochemical properties of the cosmetic product. Additionally, depending on the type of cryoprotectant used, the final viability of the probiotic in the product may vary. During the lyophilization process, exposure of probiotic strains to water should be avoided. The presence of moisture can lead to the rehydration of dried organisms, resulting in either proliferation or death. Therefore, the use of oil-based formulations may be considered a potential solution. A significant issue in this case is whether probiotics can easily be released from the oily formulation when applied to the skin and whether they can become metabolically active to exert the intended effect [97].

Another approach to preserving and extending the viability of probiotics is their microencapsulation in polymeric or lipidic particles [98]. In a study evaluating the effectiveness of the microencapsulation process in preserving the viability of probiotic bacteria in cosmetic products and preventing their inactivation by preservatives commonly used in them, alginate microspheres containing *Lactobacillus casei* were prepared. The microencapsulation process within the alginate microspheres was shown to protect probiotics against factors such as temperature, UV light, and antimicrobial preservatives. Consequently, they enhance the viability of the beneficial bacteria [102].

Another important issue encountered in the development of probiotic-based cosmetic products is the presence of antimicrobial preservatives as ingredients. Cosmetic products are not sterile, but they have to meet microbiological quality requirements and should be free from specific microorganisms, such as *Pseudomonas aeruginosa*, *Escherichia coli*, *Staphylococcus aureus*, and *Candida albicans*. Because of this, they typically contain antimicrobial preservatives to prevent microbial contamination and growth. However, these preservatives may also have a negative impact on the viability of probiotic strains [74,99].

The safety of a cosmetic product and its ingredients is a mandatory requirement under EU cosmetics regulations. Therefore, the safety of probiotics as an ingredient should be prioritized. Currently, there are no specific guidelines for the commercialization of probiotics or probiotic-containing products. The absence of regulatory guidelines that specify production requirements presents a significant challenge in assessing the quality of probiotic-based products. A safety assessment tailored specifically for probiotic-based cosmetics needs to be developed and adapted. Additional research is required to confirm the safety, efficacy, and mechanism of action of cosmetic products containing probiotics [97,98,99]. On the other hand, cosmetic products are required to have a microbial content of less than 500 colony-forming units (CFU)/g for eye-area products and less than 1000 CFU/g for other products, in terms of safety. The high colony-forming unit content in probiotic-containing cosmeceutics presents a challenge in ensuring these products meet the microbial load regulatory requirements established by legal authorities [74,97].

The use of postbiotics instead of probiotics in the formulation of biotic-based cosmeceuticals designed to regulate the skin microbiota can be considered an alternative approach that offers a potential solution to the issues mentioned above. While postbiotics provide similar benefits to probiotics on the skin, they are safer due to the fact that they are not viable and do not proliferate. Additionally, postbiotics offer advantages over probiotics, such as superior stability during production and storage, as well as longer shelf life [85].

## 4. Marine Cosmeceuticals

In recent years, natural or organic raw material contents have attracted great attention in the cosmetic industry. A significant portion of natural raw materials can be obtained from different parts of plants, such as stems, flowers, leaves, fruits, and roots. However, in organic agriculture, the slow growth rate of plants and the limited resource of arable lands restrict the processing of plant-based raw materials. Alternatively, the habitat and organism diversity of the oceans, modern aquaculture techniques, and biotechnological methods used to obtain marine raw materials have made marine resources important for the cosmetic industry. The use of existing marine resources is important not only economically for countries with sea or ocean coasts but also due to today’s consumers’ awareness of environmental protection and the use of sustainable energy sources and their interest in natural cosmeceuticals [103,104].

As an example, collagen is an important bioactive ingredient obtained from cattle and pig processing industries for use in cosmetic products. However, in addition to problems such as high cost, limited availability, the potential to trigger immune reactions, and the risk of encephalopathy in obtaining collagen from animal sources, different religious beliefs also limit the use of animal collagen. The fact that no infectious diseases have been reported related to collagen and similar compounds obtained from marine plants and animals and the absence of religious restrictions on their use have increased the interest in these marine raw materials [103].

The increasing interest of consumers in green and environmentally friendly products containing natural, safe, and effective ingredients is another reason for the increase in the production of cosmetic products prepared using marine raw materials and scientific studies on this subject. Oceans and seas contain a wide variety of sustainable plants, fish, shellfish, and microorganisms. Even sea water is considered an important cosmetic raw material [105,106]. The cosmeceutical use of natural products obtained from marine sources gained importance due to its potential as a promising source of active compounds with anti-aging effects [107]. In addition, marine polysaccharides are valuable for their use as biopolymers, which are beneficial to provide gelation in cosmetic formulations.

Therefore, in this section, the sources, classifications, and cosmetic use of marine raw materials are compiled.

### 4.1. Marine Water

Water (Aqua) is an essential ingredient for cosmetics. Even in waterless cosmetics, the production method requires water for processes such as cooling or cleaning. However, most of the formulas prepared in cosmetics still require water as a solvent. Unlike “Aqua,” sea water (Aqua Maris) can be a therapeutically effective ingredient due to its high mineral content such as chlorides, magnesium, sodium, calcium, potassium, bromides, sulfates, and bicarbonate [105,108].

Dead Sea Water is a well-known example with its high amount of vital mineral content (345 g/L). It has been reported that the amount of minerals in this water is higher than that of the oceans (39.15 g/L), and therefore it is used for therapeutic and cosmetic properties such as balneotherapy purposes together with phototherapy and as a protector against UVB-induced stress [105,108].

Sea salt contains various minerals, which can be used for preparing cosmetics such as bath salts or body scrubs and to absorb fragrances and essential oils. They have a low moisture content and can be used in skin care cosmetics for skin-lightening, deodorizing, hair conditioning, and cleansing purposes [109].

Sea mud is another marine compound that contains various minerals valuable for nutrition and cosmetics. Being rich in minerals, Dead sea mud is especially known for its therapeutic properties for psoriasis and other skin-related disorders [109].

### 4.2. Algae (Seaweed)

Algae, also known as seaweeds, are eukaryotic organisms. They can be either in macro (red and brown algae; Rhodophyta and Ochrophyta species, respectively) or in micro (green algae; *Charophyta* sp.) form. Blue and green algae types (known as cyanobacteria) are also considered prokaryotic organisms [107].

Seaweeds (red and brown macroalgae) are photoautotrophic aquatic organisms with multicellular properties that live in coastal areas. They possess the ability to perform photosynthesis, which allows them to metabolize the energy for their physiological processes. They are rich in polyphenols, sterols, alkaloids, flavonoids (phlorotannins), proteins, and essential amino acids. Also, polyunsaturated fatty acids, vitamins, and minerals, mycosporine-like amino acids (MAAs), sugars, biopolymers such as alginate and carrageenan, and pigments such as chlorophylls, xanthophyll, β-carotenoids, and fucoxanthin can be extracted from these algae [105,108,109,110,111].

On the other hand, microalgae are small unicellular or simple multicellular species that are found in various environments and can be cultivated by different biotechnological methods such as deoxidization and genetic engineering. They show high antioxidant properties due to their active ingredients such as astaxanthin, β-carotene, ascorbic acid, and tocopherol. Also, chlorophylls, lipids, and fatty acids can be synthesized from these algae [107,108,111]. All these contents of seaweeds make them valuable in pharmaceutical, food, agricultural, and cosmetic industries [111].

Alga extracts or algal ingredients can be used for skin protection and against TEWL [108,111]. On the other hand, seaweeds are considered nutrient-rich food, being rich in dietary fibers, protein, essential amino acids, polyphenols, vitamins, and minerals [112]. They are rich in vitamins (A, B1, B2, B9, B12, C, D, E, K) and the essential minerals (calcium, iron, iodine, magnesium, phosphorus, potassium, zinc, copper, manganese, selenium, fluoride) that must be taken with a daily diet. All this content makes them a beneficial choice for nutrition and, therefore, nutricosmetics.

As an example, giant kelp (*Macrocystis pyrifera*; *Phaeophyceae*), which contains many essential vitamins, minerals, and essential fatty acids such as omega-3 and omega-6, is a brown algae found in offshore areas. Kelp is used as a thickening agent in cosmetics, due to its phycocolloid content. Its phlorotannin content (phloroeckol and tetrameric phloroglucinol) exhibits antioxidant activity and UV protection. It is reported that kelp has efficacy for facilitating cell regeneration and skin health. Furthermore, extracts from kelp act like a suntan and stimulate tyrosinase activity [108,111,112].

Another example is *Fucus vesiculosus (Phaeophyceae)*, brown seaweed, which has the ability to stimulate the expression of hem oxygenase-l (HO-l). Therefore, it eliminates the hem production on the skin by removing hem catabolites. Fucus extracts can stimulate collagen production, and therefore they could help to reduce fine lines and wrinkles. Fucus extracts have been used to reduce the appearance of “eye bags” and dark circles on the skin area under the eyes due to their anti-inflammatory and antioxidant properties [112].

The *Rhodophyta* (red algae) family contains carrageenan types. Therefore, it is highly used in topical formulations, toothpaste, hair tonics, soaps, and sunscreens for its carrageenan content. Bioactive compounds from *Chlorophyta* species are also used in the cosmetics industry [112]. *Chondrus cripsus* (red algae), *Laminaria saccharina* (brown algae), *Ascophyllum nodosum* (brown algae), and *Asparagopsis armata* (red algae) are among the cosmetically important species. *Chondrus cripsus* hydrates and moisturizes skin with its high amount of polysaccharides and minerals, whereas *Ascophyllum nodosum* and *Asparagopsis armata* extracts, on the other hand, are effective on skin redness. They help to reduce the level of vascular endothelial growth factor (VEGF), which is related to the dilated capillaries, resulting in very sensitive red skin [108].

One of the best-known marine microalgae is *Chlorella*. Its extract is rich in proteins and thus can be used as a growth factor, anti-inflammatory and wound healing effects, antioxidant, and emollient. Another green algae, *Dunaliella*, contains a high amount of carotenoids. The *Dunaliella* species lives in extreme environmental conditions, which have high salinity, low pH, high irradiance, and subzero temperatures. However, it can also be cultivated in large outdoor ponds for the food industry. Their polyunsaturated fatty acid, oil, and carotenoid content make *Dunaliella* valuable for the food and cosmetic industries [108].

#### 4.2.1. Most Used Cosmetic Ingredients Obtained from Seaweeds

**Figure 1:**
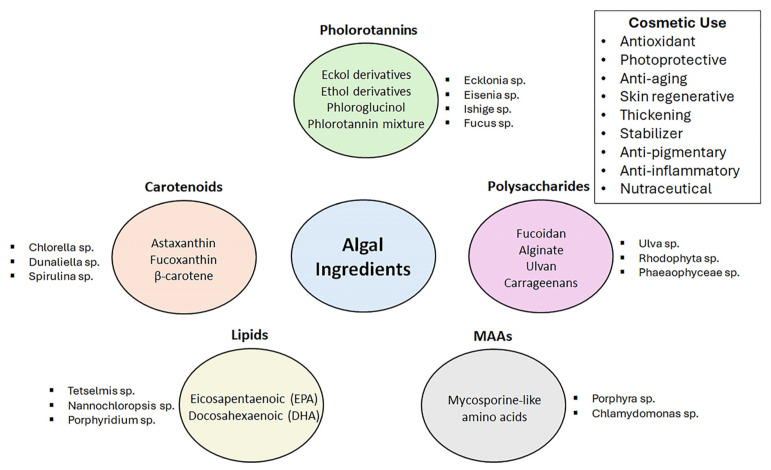
Algal ingredients and sources are frequently used in cosmetics.

Seaweeds provide many bioactive compounds that can be used in the cosmetic industry (Figure 1). They have a wide range of uses as cosmeceutical actives as photo protective against UV radiation, anti-wrinkling, antioxidant, anti-inflammatory, moisturizing, anti-allergic, anti-acne, antimicrobial, and melanin-inhibitory. Seaweed-based biopolymers such as alginate and carrageenan can also be used as thickening or water-binding agents [113,114,115].

Sulphated polysaccharides are highly found in different brown seaweeds such as *Laminaria digitate*, *Ascophyllum nodosum*, *Laminarina hyperborean*, and *Laminaria japonica*, which contain laminarins (32–35%); and *Cladosiphon* species, *Undaria pinnatifida*, *Fucus vesiculosus*, and *Laminaria japonica*, which contain fucoidans. Red algae are rich in porphyrans and carrageenans. *Eucheuma* species, *Chondrus crispus*, and *Gigartina stellate* contain 15–40% carrageenan in their cell walls and intercellular tissue matrix. Green algae (*Ulva* species) are reported to contain ulvans in their cell walls. Sulphated polysaccharides provide antioxidant, antibacterial, anti-inflammatory, and wound-healing effects [108,113].

Among these polysaccharides, laminarin is a β-glucan derivative with a β-(1-3)-linked glucan backbone and β-(1-6)-linked side chains. Laminarin is reported to have antioxidant and photo-protective activity against UVB radiation, and it affects the destruction of dermal collagen fibers by enhancing antioxidant enzymes [113,116,117].

It is also effective as a wound-healing agent. Fucoidan is another polysaccharide that has an effect against skin damage and the harmful effects of UVB exposure. It shows anti-inflammatory activity and photoprotection against UV radiation [118]. Ulvan is one of the most important seaweed polysaccharides. It consists of rhamnose, glucuronic acid, iduronic acid, xylose, and a repeating disaccharide structure comprising a uronic acid linked to a sulfated neutral sugar. It has moisturizing properties and antioxidant activity. Its ability for cell proliferation and collagen synthesis makes ulvan an interesting raw material for the cosmetic industry [108].

Macroalgal-derived phycocolloids are high molecular weight, structural polysaccharides found in the cell wall of marine algae that typically form colloidal solutions such as carrageenan, agar, and alginate [119]. Among them, carrageenans are sulfated galactans. They show water-binding, gel-forming, viscosity-increasing, and thickening properties. They can be classified as iota (ι), lambda (λ), and kappa (κ) carrageenans due to their gelling and solubility properties [120]. They are used to soften, soothe, and moisturize the skin in addition to improving the lubricity and softness of cosmetic products [113,117]. Alginate oligosaccharides are reported to enhance neonatal keratinocyte growth and can be used for EGF-dependent stimulation of keratinocytes [121].

Natural pigments are another group of ingredients that seaweeds contain in high amounts. Lipophilic essential pigments are found in brown (carotenoids), green (chlorophylls), and red (phycobilins as phycoerythrin) algae [117]. In addition to their color-enhancing properties, these compounds show free radical scavenging, melanogenesis inhibiting, and photoprotection effects on skin [106]. Astaxanthin and fucoxanthin are the most known examples. Fucoxanthin is a xanthophyll carotenoid mostly found in *Undaria pinnatifida* and *Laminaria japonica*. It shows anti-aging, antioxidant, anti-angiogenic, and anti-inflammatory properties [113]. Its photoprotective effect is the highest among xanthines due to the allenic bond and hydroxyl, epoxy, carbonyl, and carboxyl moieties found in the terminal rings. The allenic bond and 5,6-monoepoxide play an important role in the antioxidant activity of fucoxanthin [113,122,123]. Astaxanthin is also a xanthophyll carotenoid naturally biosynthesized by various algae, some yeasts, bacteria, and marine animals [124]. It is highly found in *Haematococcus pluvialis*, a single-celled alga [125]. Astaxanthin has a higher antioxidant capacity than other xanthine types except fucoxanthin. Even though it has a similar chemical structure to β-carotene, astaxanthin shows higher antioxidant capacity due to its polar ionone rings on both ends quenching the free radicals and other reactive oxygen species [113,126].

Mycosporine-Like Amino Acids (MAAs) are colorless, low molecular weight, and water-soluble molecules highly found in red *(Porphyra* sp.) and brown algae. Their chemical structure consists of a cyclohexenime ring conjugated with two amino acids, amino alcohols, or amino group substitutions [113]. MAAs have UVA and UVB radiation absorption capacity and show anti-photoaging activities [127,128,129]. They protect the skin against UV irradiation-induced damage by inhibiting the expression of the MMP gene by suppressing the MAPK signal pathway [113]. They have been reported to have multiple types of activity, such as UV radiation absorbing, anti-photoaging, and antioxidant activities, as a result of their phenolic hydroxyl structure, which can effectively dispel ROS [130]. The investigations in the literature have indicated that the anti-photoaging activities of seaweed-derived MAAs are not only mediated by their photoprotective activity by absorbing UVR but also by potent antioxidant, anti-inflammatory, radical scavenging, macromolecule damage-protection, and MMP inhibitor activities [131].

Phenolic compounds such as lignans, phlorotannins, and terpenes are also important ingredients of macro algae. Lignins are the combination of dimeric or oligomeric structures such as monolignols, coniferyl alcohol, and sinapyl alcohol, highly found in *Rhodophyta*. Phlorotannins are a chemical subgroup of tannins together with hydrolyzable and flavonoid-based tannins. Phlorotannins, oligomers of phloroglucinol, are only found in brown algae. They have greater antioxidant capacity than tocopherol or ascorbic acid and also exhibit anti-inflammatory activity. Various types of algae contain different phlorotannins such as 7-phloroeckol, eckstolonol, eckol, dieckol, dioxinodehydroeckol, and phlorofucofurofuroeckol, which can be used as whiteners and anti-wrinkle agents due to their effect as hyaluronidase and tyrosinase inhibitors. Phlorotannins from *Ecklonia cava* have also been tested for their effectiveness as antibacterial agents [119,132,133].

Flavonoids such as kaempferol, quercetin, caffeic acid, catechol, vitexin-rhamnosee, and chlorogenic acid are antioxidant compounds, which can be extracted from marine algae such as *Caleurpa* sp. *(Chlorophyta)* or *Acanthophora spicifera (Rhodophyta)* [133].

Terpenoids are another group of compounds that show antioxidant properties and can be extracted from marine algae. For example, a chromene-based phenolic compound from *Gracilaria opuntia* (*Rhodophyta*) is reported to have antioxidant properties. Diterpenes and sesquiterpenes are terpenoid compounds frequently found in red algae such as *Sargassaceae* and *Rhodomelaceae*. Sesquiterpene is also used in fragrances [133].

#### 4.2.2. Extraction Techniques for Microalgae

As microalgae-containing cosmetics are considered a growing area of research, the synthesis of new functional ingredients derived from algal compounds is also becoming increasingly important. Microalgae harvesting is usually a sequential process that involves removing water content from microalgae through a series of downstream steps to concentrate the biomass. When choosing the right harvesting method, it is necessary to consider the total cost and energy consumption, which are largely determined by cell size and density. It is also necessary to establish an excellent cell recovery technology that can be used with most microalgae species, recovers most of the microalgae biomass, and has minimal operating, energy, and maintenance costs [133].

For algae extraction, either conventional or green methods can be used. Centrifugation is the most used conventional method. Coagulation, sedimentation, filtration, and electrical techniques are other conventional microalgae harvesting methods. These techniques have some disadvantages, such as energy usage and cost in centrifugation, being unsuitable for a large diversity of microalgae in sedimentation. Although the filtering technology is efficient, it has clogging and fouling issues that can result in poor harvesting yields. The electrical method of harvesting includes electro-coagulation and flotation, which have large energy requirements for supplying and consuming the microbubbles as well as high equipment costs. All these disadvantages make conventional methods inappropriate for the harvesting process.

Instead, green technologies can also be used for algae extraction. These techniques reduce solvent waste, increase yield, automate the process, and save time and energy [133]. Supercritical fluid extraction, pressurized liquid extraction, subcritical water extraction, and microwave-assisted extraction are the best-known examples. The most frequently used one is the supercritical CO_2_ extraction. It uses a “Generally Recognized As Safe” solvent, carbon dioxide, as a supercritical fluid with temperature over 31 °C and pressure over 74 bar that is defined as a supercritical state [133,134,135].

### 4.3. Marine Microorganisms

Marine bacteria and fungi are another source of bioactive compounds that can be used in the cosmetic industry (Table 3). Marine fungi are rich in tyrosinase inhibitors such as isoflavones and pyrones, along with terpenes, steroids, and alkaloids. These may reversibly or irreversibly inactivate the enzyme [136]. Tyrosinase plays a key role in melanogenesis, and tyrosinase inhibitors have an effect on skin whitening.

Exopolymers and exopolysaccharides are important deep-sea compounds that can be used for cosmetic purposes. They are found in biofilms consisting of prokaryotic and eukaryotic microbial communities. The main components of biofilms include polysaccharides, proteins, extracellular DNA, lipids, and extracellular polymeric substances. *Planococcus maitriensis Anita I* is an important source for exopolymers. On the other hand, *Alvinella pompejana*, and *Alteromonas macleodii* subsp. *Fijiensis*, *Vibrio diabolicus*, *A. infernus*, and bacterium HYD721 are sources of exopolysaccharides. Exopolysaccharides contain glucose, galactose, rhamnose, fucose, mannose, glucuronic acid, and galacturonic acid. Therefore, they are frequently used in cosmetics [104,108].

Marine microorganisms can contain pigments or MAAs that can absorb UV rays. MAAs can be distributed in the cytoplasm of fungal cells and enhance UV protection for themselves [137]. Biopterin glucose, a marine planktonic cyanobacterium-sourced pigment that is used in sunscreen formulations against UV-A radiation is another example. Scytonemin, a carotenoid produced by marine cyanobacteria, can also be used as a UV sunscreen in cosmetics [108].

Another use of marine bacteria is to be a source of preservatives. Microbulbifer is a genus of halophilic bacteria that are commonly detected in the commensal marine microbiomes. It is known for its polymeric material and polysaccharide degrading ability. Microbulbifer was also reported to produce 4-hydroxybenzoate that can be used instead of parabens, which are prepared from 4-hydroxybenzoate. Being a petrochemical derivative, paraben use is limited in natural cosmetics. Microbulbifer A4B-17 is reported to be the first microorganism to accumulate 4HBA and parabens, most probably as a metabolite derived from the shikimate pathway [138].

Marine bacteria can produce hyaluronic acid or special enzymes to catalyze the removal reactions of free radicals caused by UV damage at high temperatures. These enzymes have been isolated from the extremophile Thermus thermophilus and are used for developing commercial products for damaged skin with UV radiation [107].

Extremophilic microbes are the types that live in virtually extreme conditions such as hot, or highly found in deep water hot springs, salt lakes, dry deserts, or volcanic areas. They can also be isolated from industrial processes such as highly basic pulp mill effluents and evaporating ponds for solar salt facilities. These microbial extremolytes are the source of low molecular weight organic molecules such as sugars, polyols, heterosides, amino acids, and their derivatives. Ectoine, hydroxyectoine, proline, mannitol, glycine-betaine, and trehalose can be named among the examples of ingredients that halophiles contain. Hyperthermophilic microbes contain heterosides, such as glycoin, glucosyl-glycerate, and firoin types. Phosphorylated, UV light-scavenging compounds, MAAs, and melanin are also isolated from extremophilic bacteria [139].

Most extremolytes protect macromolecules and cell structures of extremophiles from their hostile habitats by forming and stabilizing protective water layers around them. They are efficient chemical scavengers and prevent cells and their structures from being damaged by UV radiation and oxidative stress [139].

Ectoine (2-methyl-1,4,5,6-tetrahydropyrimidine-4-carboxylic acid), a non-proteinogenic amino acid from *Halorhodospira halochloris*, is one of the best-known halophilic microorganism-sourced active ingredients. It is an extremely halophilic phototrophic eubacterium, isolated from Wadi Natrun, Egypt. It is manufactured by fermenting a non-genetically modified organism strain of the halophilic bacterium *Halomonas elongata* (Bitop AG, Dortmund, Germany). The process is called bacterial milking. The process contains micro- or ultrafiltration, electrodialysis, chromatography, drying, and removing impurities using crystallization. In cosmetics, ectoine shields proteins, cell membranes, and human tissues from allergens, UV light, heat, and dryness and consequently has become a desired ingredient of skin and hair care products [105,139].

Some marine microorganism-sourced ingredients are given in Table 3.

**Table 3:** Some examples of bioactive ingredients sourced from marine microorganisms [140].

Bioactive Ingredient	Microorganism	Cosmetic Use
**Mycosporine** Mycosporine-glutaminol-glucoside, Mycosporine-glutamicol-glucoside	**Marine fungi** *Phaeotheca triangularis*, *Trimmatostroma salinum*, *Hortaea werneckii*, *Aureobasidium pullulans*, *Cryptococcus liquefaciens*	**Photo protective** UV screening Antioxidant UV-A screening
**MAAs** Shinorine, Porphyra-334 Mycosporine-glycine-alanine	**Marine bacteria** *Pseudonocardia strain P1*, *Micrococcus* sp. *AK-334*, *Actinosynnema mirum DSM 43827*	
**Benzodiazepine alkaloids** Circumdatins I, C, G	**Marine fungus** *Exophiala*	
**Carotenoids** β-carotene Astaxanthin Zeaxanthin Cantaxanthin Phoenicoxanthin Echinenone	**Marine bacteria** *Paracoccus and Agrobacterium* **Marine fungi** *Rhodotorula, Phaffia, Xanthophyllomyces* **Thraustochytrids** *Thraustochytrium strains*, *Ulkenia* sp., *Aurantiochytrium* sp. KH105	**Photo protective** UV screening Antioxidant Depigmentation
**Polysaccharides** EPS HE 800	**Marine fungi and bacteria** *Agrobacterium* sp., *Alcaligenes faecalis*, *Xanthomonas campestris*, *Zymonas mobilis*, *Eduarsiella tarda*, *Aureobasidium pullulans*, *Alteromonas macleodii*, *Pseudoalteromonas* sp. *Vibrio diabolicus*	**Anti-aging** Emulsifying Thickening Anti-wrinkles
**PUFAs** DHA EPA Omega-3 fatty acids	**Marine fungi** *Trichoderma* sp., *Rhodotorula mucilaginosa AMCQ8A* **Marine bacteria** *Moritella dasanensis*, *Vibrio*, *Pseudomonas*, *Shewanella and Colwellia* sp. **Thraustochytrids** *Schizochytrium*, *Aurantiochytrium*, *Ulkenia*	**Anti-aging** Soft tissue repair Skin nourishment Collagen stimulation
**Phenols** Hydroquinone derivatives	**Marine fungi** *Acremonium* sp. and *Aspergillus wentii N48*	**Antioxidant** Radical scavenging UV-A screening
**Isobenzofuranone derivatives**	**Marine fungus,** *Epicoccum* sp.	
**Exopolysaccharides** (EPS2)	**Marine fungus,** *Keissleriella* sp. YS 4108	
**Diketopiperazine alkaloids**	**Marine fungus,** *Aspergillus* sp.	
**Dioxopiperazine alkaloids**	**Marine fungus,** *Aspergillus* sp.	
**Pyrone derivatives** Kojic acid and derivates α-Pyrone derivate Phomaligol A 6-n-pentyl-α-pyrone, Myrothenone A	**Marine fungi** *Aspergillus, Penicillium*, *Alternaria species, Botrytis* sp. *Alternaria* sp. *Myrothecium* sp.	**Skin whitening** Tyrosinase inhibition
**N-acyl dehydrotyrosine derivatives** Thalassotalic acids A, B, C	**Marine Gram-negative bacterium** *Thalassotalea* sp. PP2-459	
**Dicarboxylic acid** Azelaic acid	**Marine fungus** *Malasseziales*	
**Sesqiterpenes**	**Marine fungus Pestalotiopsis sp. Z233.**	
**Alkyl halides** Methyl chloride	**Marine bacteria** *Pseudomonas*	
**Antraquinones** Chrysophanol	**Marine fungus** *Microsporum* sp.	
**Parabens**	**The marine bacterial strain** A4B-17, Microbulbifer	**Anti-microbial**
**Chitin, Chitosan**	**Marine fungi** *Zygomycetes*, *chytridiomycetes*, *ascomycetes*, *basidiomycetes*	**Anti-microbial Biopolymer Thickener**
**Protein polysaccharide complexes,** **glycolipids, lipopeptides**	**Marine fungi and bacteria** *Actinobacter*, *Pseudomonas*, *Myroides*, *Streptomyces*, *Yarrowia*, *Rhodotorula*, *Bacillus*, *Azotobacter*, *Corynebacterium*	**Emulsifying, Moisturizing**

### 4.4. Marine Animals

Ingredients obtained from marine animals are also important for the cosmetic industry. In addition to fish, corals, and crustaceans, marine animals can also be called marine animals. Extracts from fishes are the source of different vitamins, proteins, and peptides. Collagen, a structural protein, can be obtained from the fish types. Shellfish extracts also contain various elements, whereas the corals are calcium and the crustaceans are rich in chitin and astaxanthin [103,108].

*Collagen* is the most abundant extracellular matrix protein. The common feature of the collagen family, defined by their polypeptide sequences, is the superhelix conformation. Generally, a collagen structure consists of three polypeptide α chains forming a triple helical collagen. Each chain consists of simple repeating sequences of [Gly-X-Y]_n,_ where X and Y are mostly proline and hydroxyproline, respectively [108,141]. Collagen can be subdivided into fibrillar and nonfibrillar types. Fibrillary collagen types I, II, and III represent approximately 80–90% of the collagen in the human body. These collagen types are responsible for tissue strength, elasticity, and water retention. Skin fibroblasts synthesize procollagen, which is converted into collagen type I (85–90%) and type III (10–15%). In photo-aging, reduced amounts of these collagen types are a characteristic feature, mostly resulting from fibroblast aging [141]. Collagen can be obtained from the skin and bones of different animals, as well as from aquatic animals such as salmon, trout, tuna, carp, shark, tilapia, jellyfish, anemones, starfish, octopus, sea cucumber, sponge, sunfish, and sea eels [142,143,144,145]. The most important reason for using marine collagen is the risk of encephalopathy caused by bovine or porcine collagen. Porcine and bovine collagens can be highly immunogenic and inflammatory. In addition, porcine collagen and its derivatives are prohibited in Muslim communities. Instead, marine collagen presents many advantages, such as high bioavailability, being chemically inert, low antigenicity, high metabolic absorption, biodegradability, and low toxicity [141].

Collagen is a highly cross-linked substance that is normally insoluble in water and oils. Therefore, hydrolyzed collagen forms obtained by thermal processing above 40 °C are preferred in cosmetics. These water-soluble small peptides and short polypeptides are obtained by separating the α chains of collagen and then using proteolytic enzymes to perform hydrolysis. The composition and degree of hydrolysis of collagen improve functional properties such as antioxidant capacity, anti-aging and antimicrobial activity, and bioavailability [103,141,145]. Collagen is mostly used in the form of hydrolysate as a moisturizing and emollient agent in serums, masks, and fillers. Its film-forming properties and high molecular weight make collagen hydrolysate useful for reducing transepidermal water loss (TEWL) and moisturizing the skin. The skin becomes smoother, and its radiance increases. In addition, hydrolyzed collagen protects the skin from stress and premature aging [141]. It has been reported that collagen hydrolysates obtained from milkfish (*chanos*) scales exhibit significant moisture absorption, anti-skin aging, and anti-melanogenic capacities [146]. Similarly, the moisture retention and absorption capacities of collagen from sea cucumbers (*Holothuria cinerascens*) were compared with glycerol, and an efficiency of up to 72.2% was obtained [142]. Jellyfish (*Rhopilema esculentum*) collagen hydrolysate has been shown to increase the protective effect on the photo-aging of mouse skin induced by UV irradiation [147].

#### 4.4.1. Coral

Coral is a combination of animals, plants, and minerals, mainly composed of calcium. Hard coral types have a rigid calcium skeleton with elongated polyps to capture sunlight and food particles. Soft corals have polyps in varying sizes called spicules that have a soft or leathery feel. These spicules use small amounts of calcium to give them rigidity [148]. Marine gorgonian corals are rich in bioactive terpenoid compounds. As an example, Pseudopterogorgia elisabethae is the source of diterpene glycosides known as pseudopterosins. In addition to their effect in preventing sun damage and nourishing the skin, it has anti-inflammatory activity. Pseudopterosins can also provide membrane stabilization [108]. Due to these properties, they are incorporated into the skin care preparations for photo-shielding, antioxidant, anti-aging, and anti-acne properties. They can also be used as mineral suppliers due to their mineral content [149].

#### 4.4.2. Crustaceans

Crustaceans such as mussels, crabs, crayfish, lobsters, and shrimps are known as the subdivision of arthropods. Crustacean wastes are rich in various bioactive substances such as diguanosine-tetra-phosphate, chitin, chitosan, and astaxanthin. Among these components, diguanosine-tetra-phosphate is obtained from Artemia salina and is a precursor of ATP. It activates the G protein, a protein necessary for cellular signaling, and ultimately protects skin cells from environmental stress. This compound stimulates hair cell growth by increasing proliferation and oxygen consumption and reducing anoxia caused by lack of oxygen [108].

Chitin is a structural component of both terrestrial and aquatic organisms. It forms a part of the exoskeleton of crustaceans, molluscs, and arthropods. It is also found in the cell walls of many fungi and algae. Chitin obtained by processing shellfish from crustaceans such as crabs, lobsters, shrimps, krill, oysters, and marine fish like squid can be used in different industries. Due to its amino-polysaccharide structure consisting of N-acetyl-D-glucosamine residues joined by β-(1-4) bonds, chitin is insoluble in water but relatively stable under slightly acidic or basic conditions. Shell waste is initially treated with a dilute acid to remove salts and minerals, and then the sample is subjected to several treatments with hot alkaline solutions to remove remaining organic compounds. The residue left behind is then dried, and chitin is obtained as colorless or off-white flakes or powder. It is one of the most widely used biopolymers in nature. It has versatile applications in cosmetic products as a smoothening, moisturizing, and cleaning agent [105,108,150].

Chitosan is a large, cationic copolymer that is a partially deacetylated form of chitin. It is a linear polysaccharide consisting of a mixture of D-glucosamine and N-acetyl-D-glucosamine monomers joined by β-(1-4) bonds. Commercial chitosan types are characterized by their N-acetylation degrees ranging from 5% to 30%, molecular weights ranging from 100 to 1000 kDa, and can exist in amorphous, semicrystalline, or crystalline forms. The pure microcrystalline form of chitosan (MW: 10~300 kDa) had a large adsorption area. It can be used to form gels. The pKa value of the amino groups of chitosan is around 6.5, which acquires a positive charge and therefore dissolves in acidic conditions. Due to these properties and its ability to interact with various biomolecules, chitosan has several commercial applications in the areas of bioadhesives, waste treatment, food preservatives, food additives, pharmaceuticals, nutraceuticals, and cosmetics [105,108,150].

Glucosamine is an amino-monosaccharide that is widely found in marine organisms, especially in the exoskeletons of crustaceans and other arthropods and is obtained by the enzymatic hydrolysis of these exoskeletons by chitinase and chitobiase. Glucosamine and its derivative N-acetyl glucosamine are substrate precursors for the biosynthesis of polymers and proteoglycans. Glucosamine is a substance considered safe in cosmetics. It accelerates wound healing by stimulating the biosynthesis of hyaluronic acid, improves skin moisture, and reduces wrinkles. Glucosamine also exhibits anti-inflammatory and chondro-protective effects. N-Acetylglucosamine is a different form of glucosamine that acts as the building blocks of joint tissues and other connective tissues [108].

Astaxanthin is a carotenoid found abundantly in marine microorganisms and animals, including bacteria, salmon, and crustaceans. Astaxanthin has antioxidant activity, is a vitamin A precursor, regulates the immune system, and has antitumor activities on human health [108].

### 4.5. Cosmetic Use of Marine Ingredients

The use of marine ingredients as cosmetic raw materials has been gaining increasing interest recently. Marine raw materials obtained using biotechnological methods are used for their cosmeceutical effects, such as hydrating, moisturizing, anti-wrinkle, skin whitening, antioxidant, and anti-aging.

As an example, carotenoids are highly used ingredients in the cosmetic industry. They can be extracted from different marine resources such as cyanobacteria and algae and have anti-aging properties due to their ROS-preventing capacity. Another example is that exopolysaccharides are effective at reducing skin irritation against UVB aggregation due to their galacturonic acid content [106]. MAAs are also effective in ROS prevention. Helioguard^®^ 365 is a commercial example (Mibelle Biochemistry group, Buchs/Switzerland) containing porphyra-334 and shinorine extracted from red algae [105].

The major cosmetic groups use marine ingredients as a part of their formulations. To exemplify, Biotherm by Blue Therapy, Elemis by La Mer, Oceanwell by OceanBasis, Guam algae by Lacote, La Prairie, and Resilience by Estée Lauder contain marine-derived cosmeceutical products. Some other producers who manufacture commercially available marine raw materials are listed in Table 4.

#### Marine Ingredients as Biopolymers

Many marine ingredients show the ability to produce gels and are therefore used as gelling or thickening agents besides their anti-aging and active cosmeceutical effects. Gels are highly used products for skin and hair care in the cosmetic industry. They are known to increase the moisture of the skin, increase the skin feel, affect the textural properties of cosmetic products, and affect the thickness and physical stability of cosmetic products. Emulgels (cream gels) are good examples in which stability can be increased by the addition of a gelling agent into the aqueous phase of the emulsion [151]. Marine, especially algal, ingredients can be used as biopolymers to provide gelation, thickening, and viscoelasticity properties in gel-type formulations due to their physicochemical structure.

Gels are viscoelastic semi-solid systems formed by polar or non-polar liquids with the presence of a gelling agent. Among the gel types, organogels or oleogels can be prepared with organic solvents such as alcohol or oils. The gelling agents to produce these formulations are called organogelators. The gelling agents, or gelators, can also form a bigel, a class formulated from the combination of organogel and hydrogel [152]. The best-known gel types used in the cosmetic industry are hydrogels produced from a gelator and water. They have three-dimensional polymeric networks held together by cross-linked covalent bonds and weak cohesive forces in the form of either hydrogen bonds or ionic bonds. Due to the presence of hydrophilic functional groups on the biopolymer, water molecules can migrate into the polymeric network, which results in hydrogel expansion and occupation of a larger volume, which is called swelling. The most important feature of biopolymers in cosmetics comes from the ability of their swelling property in suitable solvents and their ability to mimic the three-dimensional extracellular matrix environment in natural tissues [150,152,153]. They can also be used in the preparation of microcapsules and microparticles as cosmetic carrier systems. The use of bioadhesive hydrogels for skin care can provide a long residence time on the application site. One of the biopolymer-based hydrogels used in cosmetics is the “beauty mask”, claimed to hydrate skin, restore its elasticity, and promote anti-aging performance [151,153].

Hydrogels used in cosmetic preparations can be based on numerous marine biopolymers, such as collagen, gelatin, alginate, chitosan, laminarine, and carrageenan. One of the main sources of hydrogel-forming polysaccharides is seaweed [153]. Algae-extracted polysaccharides form hydrogels through physical cross-linking, that is, noncovalent bonding that relies solely on weak interactions such as hydrogen bonding, van der Waals forces, and electrostatic interactions leading to a reversible gel formation.

Alginate and derivatives such as alginic acid can be extracted from the cell walls and intercellular spaces of brown algae, forming gels via an ionic complexation. The G blocks in alginate polysaccharide provide stiffness while the M regions are responsible for flexibility. A combination of G and M blocks in the structure and the differences in polysaccharide composition affect the gelation and final hydrogel properties [153]. Alginate hydrogels have been studied as alternative injectable materials to hyaluronic acid [151].

Another algal biopolymer, λ-Carrageenan is a linear polysaccharide composed of branched galactose substitutes and has a similar gelation mechanism to alginate. Even though it is reported to provide a gelation based on trivalent cation complexation with Fe^3+^ and Al^3+^, carrageenans are generally used as stabilizing agents and thickeners in cosmetic formulations because of their high hydrophilicity, mechanical strength, biocompatibility, and biodegradability [151,153].

Ulvan is a sulfated polysaccharide mainly composed of glucuronic acid, iduronic acid, rhamnose, xylose, mannose, glucose, and galactose. Ulvan provides a thermoreversible gel in the presence of boric acid and divalent cations such as Ca^2+^ [105,108,153].

Hydrogels can also be produced from laminarin [117]. Laminarin is a β-glucan derivative, a marine polysaccharide also reported to show antioxidant and photo-protective activities. Tumen Erden et al. (2021) produced laminarin hydrogels for their fibroblast activity and re-epithelization properties against wounds [154]. Costa et al. (2020) reported that laminarin is a non-toxic, degradable marine polysaccharide found in brown algae and is a promising sustainable polymeric candidate. Laminarin derivatives are ideally placed to enable gelation by photo-cross-linking [150].

Chitosan, a natural cationic polyelectrolyte copolymer derived from chitin, is a biodegradable natural polymer. It offers great potential for cosmetic applications due to its biocompatibility, high charge density, non-toxicity, and mucoadhesion. In cosmetic formulations, chitosan is used in the production of different products such as mascara, hair conditioner, hair mousse, and body creams due to its gel-forming properties. Chitosan hydrogels applied to the skin can form films to increase the water content in the upper skin layers. Chitosan hydrogels can be obtained by covalent cross-linking using UV radiation, and ionic and chemical cross-linking agents. The moisturizing properties of chitosan hydrogels depend on the molecular weight of chitosan and the degree of deacetylation [151]. It is reported that chitosan hydrogel-encapsulated extracellular vesicles were also effective as skin anti-aging as they promoted dermal fibroblast cell proliferation and prolonged the release of extracellular vehicles [155,156].

Collagen is a highly cross-linked material usually insoluble in both water and oils. It is a structural protein that is found in skin in the form of type 1 and type 3. These collagen types constitute parts of fibrillar structures responsible for tissue architecture and integrity. In cosmetic applications, type I collagen is prevalently used. Collagen extracted from marine sources such as fish skin and scales, marine sponges, or jellyfish umbrellas is a safer choice with comparable effects on the skin pH, moisture, and sebum rather than bovine collagen. Collagen can also be produced through fermentation in the presence of bacteria, such as *Methylobacterium*, *Solibacter usitatus*, and *Rhodopseudomonas* palustris [151,156].

Collagen can also be used in the form of gelatin, which is obtained by denaturation of proteins. Gelatin is a mixture of water-soluble proteins that binds more water than collagen. As it is partially degraded, more active groups are exposed to interactions with water via hydrogen bonds. Both collagen and gelatin are highly used in the cosmetic industry to prepare hydrogels. Hydrolyzed collagen form, which contains shorter peptides and polypeptides, is mainly used in cosmetic formulations due to collagen insolubility. Hydrolyzed collagen is well soluble in water and can be easily incorporated into several cosmetic formulations. Hydrogels made of collagen are usually prepared by the freeze-drying technique. Self-aggregation of water-dispersible collagen under freeze-drying conditions is appropriate for creating collagen hydrogels and aerogels for pharmaceutical and cosmetic applications [151,156].

The biopolymers from marine sources are available in different cosmetic formulations such as soaps, shampoos, sprays, hydrogels, emulsions, and emulgels as thickening, gelling, and texturizing agents to control viscosity and viscoelasticity and increase the physical stability of cosmetic products [117].

### 4.6. Challenges in Marine Cosmeceutical Production

Natural cosmetic products have gained importance day by day, as they promise eco-labels and wellness to the consumer. From this perspective, marine products have received increased attention. They contain a wide variety of molecules, either used as extracts or derived from marine sources via biotechnological methods. Marine algae, fish, and other marine animal species, even sea water and mud containing essential elements, together provide a huge variety of natural ingredients. Marine bacteria and fungi are the least investigated area, but they also gained importance for their cosmetic applications [140].

Sustainability is the most fundamental problem faced by marine sources used in cosmetics. Production capacity, ecotoxicity, and waste production can be a problem in the production of marine components [149,150].

In terms of capacity, it is seen that most marine resources are small in quantity, difficult to isolate, and affected by changes in environmental conditions. For example, although macroalgae have many species, access to biomass is limited for some of these species. In addition, access to marine organisms living on the deep ocean floor is also limited [149]. For this reason, natural resources are cultivated and harvested in bio-refineries, and then the relevant compounds are obtained by hemi-synthesis [150]. For example, monospecific cultures can be obtained from macroalgae cells selected from a single drop of seawater. Therefore, creating a bank of macroalgae cell strains with commercial value in terms of cosmetics is a strategic approach. In this way, standardization of the marine component can also be achieved [157].

On the other hand, using marine by-products as a resource of cosmetic ingredients can be effective in the reduction in marine pollution. The fish processing industry produces thousands of tons of by-products containing valuable biocomponents such as hydrolyzed collagen and hyaluronic acid during the transformation of fish or seafood. The transformation of these by-products into high value-added products not only provides natural raw materials from sustainable sources but also contributes to the reduction in marine pollution caused by fish processing industry waste [158]. Similarly, the use of marine-derived UV filters or antibacterial agents can reduce marine pollution caused by synthetic versions of the ingredients [137,138].

Compounds obtained from the sea can be used in many areas, such as medicine, the food industry, and cosmetics. When it comes to skin care, these raw materials are not only included in skin care products for cosmetic and cosmeceutical purposes but are also used as nutraceuticals or nutricosmetics due to the lipids, vitamins, and essential elements they contain. Therefore, their safety, efficacy, and quality requirements must be understood and characterized in the choice of the relevant product category during legal applications [149]. One of the issues of legal importance and difficulty in terms of marine raw materials is the toxicological evaluation of heavy metals such as cadmium, lead, arsenic, and mercury, as well as toxins and allergens naturally found in the marine ecosystem [149]. From the safety perspective, it is necessary to know the name of the alga from which the ingredient is derived, the method used for extraction, the main composition of the extract, the identity of any preservatives, additives, or contaminants, and microbiological quality [105].

Another concern about the use of marine raw materials in personal care products is the pollutants that are spilled into the sea. Plastics are the main pollutants in the sea and constitute more than 50% of marine litter. These pollutants in the ecosystem threaten not only the marine ecosystem but also indirectly human health due to components such as microplastics. Oil spills, herbicides and pesticides, and radionuclides concentrated by marine biological species can also pollute the sea. A study conducted by the United States Environmental Protection Agency reported that the presence of inorganic pollutants such as copper, arsenic, aluminum, iron, manganese, cadmium, lead, and zinc in the marine environment has the potential to pose a risk to human health [105,149,150]. Biological contamination from aquatic and air pollution and the accumulation of nutrients, salt, and other organisms in the blind angles of bioreactors used for microalgae cultivation can also be named a challenge [150].

Using sustainably produced natural-source materials for packaging is a strategy against environmental pollution. For example, seaweed is considered a zero-waste packaging material because it can be converted into packaging without the need for chemicals. Efforts are being made to reduce marine pollution and eco-toxicity concerns caused by packaging with zero-waste and edible packaging that can be obtained this way [149].

## 5. Future Perspectives

As discussed comprehensively above, the use of peptides, biotics, and marine ingredients in cosmeceutical products has gained significant popularity due to their numerous potential benefits. However, alongside their advantages, some properties of these ingredients can limit or complicate their use. The primary goal of future research should be to develop strategies to overcome these limitations. Extensive formulation development efforts are necessary to preserve the structural stability and biological activity of cosmeceutical peptides, as well as to enhance their skin penetration. In the realm of micro-ecological skin care, maintaining the viability of probiotics from production to consumer use is a significant challenge for the cosmetic industry. The encapsulation strategy within suitable carrier systems is an area that warrants extensive research. Another potential solution could be the use of postbiotics instead of probiotics, offering a safer and more stable alternative. Eco-labeled and natural products containing marine ingredients are in high demand by consumers. However, the limited quantity of most marine resources and the difficulties associated with isolating and standardizing marine components pose significant challenges. Sustainable and standardized production of marine ingredients is an important topic that will need comprehensive research in the future. Furthermore, a future focus should be aimed at developing effective, safe, and stable formulations that combine peptides, biotic, and marine ingredients. The formulation of synergistic cosmeceutical products containing peptides, biotics, and marine components will enable the creation of more effective, natural, and multi-functional cosmeceuticals for consumers.

## 6. Conclusions

In recent years, the development of cosmeceutical products containing peptides, biotic ingredients, and marine-based compounds has become a highly popular strategy to enhance anti-aging effects and better address consumer demands. The number of scientific studies and commercially available products in this field continues to grow. Promising findings from scientific research highlight the enhanced anti-aging and skin-improving properties of products containing peptides, biotic, and marine ingredients. On the other hand, challenges remain in the formulation design of these products. In conclusion, it is undeniable that more comprehensive investigations are required to evaluate the efficacy, safety, and stability of peptides, biotics, and marine-based products in preventing skin aging.

## Figures and Tables

**Table 1:** Some examples of commercial cosmeceutical products with peptide ingredients throughout the world [54].

Product	Peptide Ingredient	Claim
Dēpology Matrixyl^®^3000 Collagen Boosting Serum, Dēpology, Seoul, Republic of Korea	Palmitoyl Tripeptide-1 Palmitoyl Tripeptide-5 Palmitoyl Tetrapeptide-7	Hydrates the skin, and reduces the appearance of fine lines and wrinkles.
Eva Naturals Matrixyl^®^3000 Argireline Vitamin C Serum, Eva Naturals, LA, USA	Argireline Palmitoyl Tripeptide-1 Palmitoyl Tetrapeptide-7	Boosts collagen production for reduced wrinkles and firmer skin.
Skingenics Youthful Boost Serum, Spain	Palmitoyl Tripeptide-1	Brightens and balances overall skin tone.
Clinique 2-in-1 Cleansing Micellar Gel + Light Makeup Remover, Estée Lauder Companies, USA	Palmitoyl Tripeptide-1 Palmitoyl Tetrapeptide-7	Leaves skin feeling comfortably hydrated, refreshed, and residue-free.
Jeuvenile Multi Peptide Copper Peptide %3 Serum, Jeuvenile, France + Turkey	Copper Tripeptide-1 Argireline Palmitoyl Tripeptide-1 Palmitoyl Tetrapeptide-7	Supports skin renewal and firmness, strengthens the skin barrier, and prevents the appearance of fine lines and wrinkles.
JKOSMEC Skin Solution Peptide Mask, JKOSMEC, Republic of Korea	Palmitoyl Pentapeptide-4 Palmitoyl Tripeptide-1 Tripeptide-1 Copper Tripeptide-1 Hexapeptide-9	Maintains skin elasticity.
Estée Lauder Pure Color Envy Color Replenish Lip Balm, Estée Lauder Companies, USA	Palmitoyl Tripeptide-1	Moisturizes lips with a touch of natural color.
Elizabeth Grant Suprême Cell Vitality Victory Eye Serum, Elizabeth Grant Skin Care, Toronto, ON, Canada	Palmitoyl Pentapeptide-4	Reduces the appearance of under-eye puffiness, and restores skin radiance.
NutriBiotic Antioxidant Properties Hydrating Facial Toner, NutriBiotic, CA, USA	Palmitoyl Pentapeptide-3 Acetyl Hexapeptide-3 (Argireline)	Hydrates and refreshes skin for a healthy appearance.
Advanced Clinicals Peptide Serum, Advanced Clinicals, Midwest USA	Argireline Palmitoyl Tripeptide-1 Pentapeptide-18 Palmitoyl Tetrapeptide-7	Improves skin elasticity and hydration, and increases skin brightness.
Le Mieux Eye Wrinkle Corrector, USA	Acetyl Octapeptide-3 Argireline Acetyl Tetrapeptide-5 (Eyeseryl) Palmitoyl Pentapeptide-4 Palmitoyl Tetrapeptide-7 Palmitoyl Tripeptide-1	Helps firm and lift eye contours, minimizes the look of fine lines and wrinkles, and reduces the appearance of dark circles and puffiness.
Yves Rocher Sensitive Camomille, France	Tripeptide-1	Moisturizes sensitive skin.
Missha Time Revolution Night Repair Ampoule Gold, Able C&C Co., Seoul, Republic of Korea	Argireline Copper Tripeptide-1 Tripeptide-1	Repairs damage, evens skin tone, and improves the health of skin.
Missha Time Revolution Immortal Youth Blue Essence, Able C&C Co., Seoul, Republic of Korea	Tripeptide-1	Reduces the signs of aging and brightens the appearance of skin.
Neova Serious Clarity 4× Complexed Brightening Serum, Neova Smart Skincare, New Jersey, USA	Manganese Tripeptide-1	Renews skin clarity and reduces the appearance of discolorations.
NCN PRO SKINCARE Multi-Active Accelerator, USA	Matrixyl Palmitoyl Tripeptide-1 Palmitoyl Tetrapeptide-7 Palmitoyl Tripeptide-38 Soy-Rice Peptides (Hydrolyzed Rice Bran Protein, Soybean Protein)	Stimulates collagen production, helps firm skin, and decreases wrinkles.
Elizabeth Arden Prevage Anti-Aging Eye Serum, Revlon, USA	Acetyl Tetrapeptide-5 Palmitoyl Oligopeptide Palmitoyl Tetrapeptide-7 Palmitoyl Tripeptide-5	Helps correct the skin’s appearance.
The Ordinary Multi Peptide Eye Serum, DECIEM | Esteé Lauder Companies, Canada+USA	Acetyl Tetrapeptide-5 Palmitoyl Tripeptide-38 Myristoyl Nonapeptide-3	Reduces the signs of aging around the eyes.
Estée Lauder Futurist Power Peptide Serum Primer, Estée Lauder Companies, USA	Argireline Palmitoyl Hexapeptide-12 Whey Protein Soybean Protein	Plumps skin with hydration, and smoothes the look of fine lines.
Neostrata Skin Active Firming Collagen Booster, USA	Palmitoyl Hexapeptide-12 (Biopeptide EL) Palmitoyl Tetrapeptide-7	Enhances and preserves natural collagen for tighter and firmer skin.
L’Oreal Youth Code Ferment Pre Essence, France	Palmitoyl Hexapeptide-12 (Biopeptide EL) Palmitoyl Tetrapeptide-7	Smoothens and softens skin, and improves the skin texture.

**Table 2:** Some examples of commercial cosmeceutical products with biotic ingredients throughout the world [74,96].

Product	Biotic Ingredient	Claim
Clinique Redness Solutions Daily Relief Cream, Estée Lauder Companies, USA	*Lactobacillus*	Restores skin barrier, reduces redness and irritation of the skin.
Clinique Redness Solutions Makeup Broad Spectrum SPF 15 With Probiotic Technology Foundation, Estée Lauder Companies, USA	*Lactobacillus* ferment	Covers flushing and redness, protects against UV radiation
Missha Time Revolution The First Treatment Essence Rx, Able C&C Co., Seoul, Republic of Korea	*Bifida* ferment lysate	Promotes the moisture retention and the elasticity of the skin.
Elizabeth Arden Superstart Probiotic Boost Skin Renewal Biocellulose Mask, Revlon, USA	*Lactococcus* ferment lysate, inactivated strains of *Lactobacillus casei* and *Lactobacillus acidophilus*	Optimizes the skin microflora, moisturizes and smoothes the skin.
Andalou Naturals Brightening Probiotic + Vitamin C Renewal Cream, USA	*Bacillus coagulans*	Enzymatically supports dermal vitality and provides a tighter- and brighter-looking appearance.
Okana Probiotic Eye Serum, New Zealand	*Bacillus* bacterial ferment extract	Brightens under eyes, reduces the eye-bags and wrinkles, and lifts the eyelids.
Mother Dirt AO + Mist, USA	*Nitrosomonas eutropha*	Maintains healthy skin by balancing skin microbiota, and provides more hydrated and less-oily skin.
FCL Pre+ Probiotic Body lotion	Probiotic *bifido* cultures	Improves the barrier function of the skin, provides the balance of skin microbiota, and enhances skin hydration.
Korres Greek Yogurt Skin nourishing probiotic gel-cream, Greece	*Lactobacillus*, Greek yogurt, yogurt powder	Nourishes and hydrates the skin.
Korres Probiotic Superdose Facemask, Greece	*Lactobacillus*, Greek yogurt	Replenishes the skin, restores the microbiota.
Beekman 1802 Milk Drops Probiotic Ceramide Serum, USA	*Bifida* ferment lysate	Moisturizes and restores the skin, cleans the pores, and helps to control oil secretion.
Beekman 1802 Milk Bar Probiotic Facial Cleansing Bar, USA	*Bifida* ferment lysate	Deeply cleanses skin with ultra-foaming lather.
Pacifica Sun + Skincare Mineral Face Shade Coconut Probiotic SPF 30, USA	*Lactococcus* ferment lysate	Provides water-resistant protection with a sheer application.
WLDKAT Prebiotic + Probiotic Pleasure Serum, USA	*Lactobacillus* ferment	Maintains natural moisture of the vagina, and regulates the pH balance.
Boscia Prebiotic + Probiotic Freshening All-Over Body Deodorant, Japan + USA	*Lactococcus* ferment lysate	Neutralizes odor, restores skin balance.
Aveeno Daily Moisturizing Oat Body Wash for Normal to Dry Skin, Johnson & Johnson, USA	Prebiotics—oat flour, oat extract, oat oil	Gently cleanses the skin, and preserves the natural moisture barrier of skin.
La Roche Posay Effaclar Duo (+), France	*Aqua Posae filiformis* *(Vitreoscilla filiformis)*	Reduces the appearance of blemishes and blackheads, controls shine.
La Roche Posay Lipikar Syndet AP+, France	*Aqua Posae filiformis* *(Vitreoscilla filiformis)*	Immediately soothes and reduces severe dryness starting from the shower.
Lancome Advanced Génifique Youth Activating Serum, France	*Bifidobacterium longuum*, *Lactobacillus*, yeast, alpha-glucooligosaccharide, beta-fructooligosaccharide, mannose	Builds up the skin’s recovery, strengthens the skin barrier, and increases skin hydration.
Lancome Advanced Génifique Yeux Light-Pearl™ Serum, France	Yeast and Bifidus	Ensures radiant skin, prevents the loss of firmness, reduces the appearance of under-eye bags, crow’s feet, wrinkles anddark circles.
SK-II Pitera™ Facial Treatment Essence, Procter & Gamble, USA	Galactomyces ferment filtrate (PITERA™)	Reduces the appearance of dark spots and redness, and improves the surface cell renewal rate.
LACTOClear Blemish Clear Spot Ampoule, Cell Biotech International Co., Denmark	*Enterococcus faecalis* CBT SL5	Balances moisture and sebum levels of acne-prone and sensitive skin.

**Table 4:** Some commercial examples of marine biotechnology ingredients and their use in cosmetics.

Marine Ingredient	Source	Cosmetic Purpose	Producer
Marine Hydrolyzed Collagen lmw™ Marine collagen oligopeptides	Upcycled from fish skin	Biofunctional ingredient for scalp and hair	Ashland Global Holdings (Wilmington, DE, USA)
Celtosome™ *Crithmum maritimum* callus	Halophyte plants	Anti-stress conditions such as UV exposure	Seppic Pontrieux (Brittany, France)
Celebrity™ Technology	Macroalgal cell culture	Nutraceuticals and cosmetics	
CONTACTICEL™ Hydrolyzed Rhodophyceae Extract	Acrochaetium Moniliforme (epiphytic macroalgae)	Anti-pollution	
B-Lightyl™	*Himanthalia elongata* brown macro-algae	Hyperpigmentation disorders and dark spots eraser	Givaudan S.A. (Zurich, Switzerland)
Depollutine^®^ A peptidic extract with arginine and pyrrolidone carboxylic acid	*Phaeodactylum tricornutum* microalgae	Anti-pollution agent	
Eau de Source Marine Manganese and silicium	Spring sea water found in Brittany	Moisturizer	
Gravityl™	Gigartina stellata red macro algae	Facial lifting and reshaping	
Hydrintense™	*Porphyridium cruentum* red microalgae	A skin moisturizer resists dehydration	
Mariliance™	*Rhodosorus marinus* red microalgae	Neuro-soother	
Megassane^®^ *Lipidic extract*	*Phaeodactylum tricornutum* microalgae	Detoxifier and highlights skin tone by proteasome activation of cells	
Pro-DG™	*Dysmorphococcus globosus* microalgae	Slimming revolution	
Sensityl™	*Phaeodactylum tricornutum* microalgae found in coastal marine	Soothing, calming, rebalancing effects on sensitive skin and skin microbiota	
Wakamine^®^ 1% or XP	*Undaria pinnatifida* brown macroalgae	Skin lightening	
Seacode™	Pseudoalteromonas Ferment Extract	Anti-wrinkle enhances dermal proteins to reduce aging signs	The Lubrizol Corporation (Surrey, UK) (Formerly Lipotec SAU-Barcelona, Spain)
Hyadisine^®^	Pseudoalteromonas Ferment Extract	Anti-dehydration and anti-wrinkle	
Antarcticine^®^ marine ingredient C A glycoprotein exopolymer	Isolated from strain of Pseudoalteromonas Antarctica NF3	Skin protection from extreme cold, collagen stimulation, firmness, and elasticity increase, and reduce wrinkles	
Cellynkage™ Saccharide Isomerate (exopolysaccharide)	The microorganisms living in the Agua Amarga salt marsh	Enhances the communication of skin cells to rejuvenate mature skin	
Hyanify™ Exopolysaccharide	Isolated from the surface of Laminaria algae	Hyaluronic acid synthesis stimulation, skin replenishment	
HelioPure^®^ Skin concentrated form of fucoxanthin	*Phaeodactylum tricornutum* microalgae stabilized in jojoba oil	Photoaging protection	Algatech; Solabia Group (Paris, France)
Invincity^®^ and Invincity^®^ Powder, concentrated form of high molecular weight fucoidans	Intracellular polysaccharides of *Ascophyllum nodosum* brown algae	Fights against dehydration, dark spots, and redness, and protects the skin from the damaging effects of pollution	Algues and Mer; Solabia Group (Paris, France)
SeaLight^®^ concentrated form of sulfated arabinogalactans	They are obtained from green algae Codium fragile	Skin detoxification, microcirculation stimulation, ATP production increase	
AstaPure^®^ Skin concentrated form of astaxanthin	*Haematococcus pluvialis* micro-algae	Antioxidant	
Essenc’Age^®^ composed of fucoidans, sulfated polysaccharides	*Ascophyllum nodosum* brown algae	protects the telomeres and has a senostatic effect, stimulates cellular metabolism, reduces inflammation	
PHYCOSACCHARIDE AI Alginate oligosaccharide	*Laminaria digitata* brown algae	Acts on inflammatory mediators and recruitment of epidermal stem cells to repair and soothe damaged skin	Codif Technologie naturelle (Saint-Malo, France)
DERMOSCULPT GMNB Contains vegetable proteins, and amino acids including the essential types	*Chlorella vulgaris* green micro-algae	Increases cell growth factors and GAG components. Boosts collagen synthesis	
EPIDERMIST Marine ExoPolySaccharide	Isolated from *Vibrio* sp. by biotechnology	Improves skin texture and barrier. Reduce inflammatory processes	
The Maritech^®^ Fucoidan and marine polyphenols	Fucoidan naturally occurs in the cell walls of brown seaweeds	Nutricosmetics and beauty-from-within	Marinova Pty LTD (Tweed Heads, Australia)
Algaenia^®^ Chlamydomonas acidophila extract rich in peptides	Chlamydomonas acidophila green microalgae	Soothes, prevents sensitization, moisturizes, modulates inflammation against stress	Laboratoires Expanscience (Paris, France)
VIVASTAR^®^ alginates ALGOGEL^®^ RCG carrageenans ALGANACT™ agri-ingredients	Laminaria digitate Lamanaria hyperborean *Ascophyllum nodosum*	A range of biopolymers from Seaweed-based alginates, carrageenans and agri-ingredients	Algaia (formerly Eviagenics)- Lannilis, France
Affinisphere™ Marine Atelocollagen microspheres	Shark fin	Improvement of oily skin	BASF (Ludwigshafen, Germany)
Marine Filling Spheres™ dehydrated microspheres of marine collagen and glycosaminoglycans	Contains atelocollagen obtained from shark fin	Moisturization, anti-wrinkle	
Sea Weed Herbasec^®^ Sea Weed Herbasol^®^ Extract PG(PF)	*Fucus vesiculosus* L.	Anti-aging, antioxidant, detoxification, moisturizing, soothing	Lipoid Kosmetik AG-(Steinhausen, Switzerland)
PADINAMI™ EC	Biofermentation of Pseudoalteromonas strain	Reinforces the skin’s youthfulness due to its collagen, GAG, and HA booster benefits	Croda Beauty (Yorkshire, UK)
Luceane™ Bioactive saccharide extract	Biofermentation of marine bacteria Pseudoalteromonas	Acts on skin holobiont to reinforce barrier function and boosts cell energy to combat *hypoxiageing™* increased by pollution. Anti-aging and revitalizing	
COLLASURGE™ Collagen Amino Acids	Marine collagen	Moisturizing, hydrating, nourishing, repairing, soothing, and softening	
CRODAROM^®^ BLACK PEARL consist of aragonite and conchiolin	Tahitian black pearls	Revitalize, moisturize, nourish, and protect skin against environmental damage	
CRODAROM^®^ SEAFOAM contains magnesium and silicon	Black Sea seafoam	Reduces the stress on skin cells and contributes to the structure and elasticity of the skin	
PADINAMI™ EC	Padina pavonica, brown algae, found in the Mediterranean, the Black Sea, the Red Sea, and oceans	Promote glycosaminoglycan renewal, stimulate the sulfated GAGs and hyaluronic acid synthesis, and boost collagen synthesis. Improves cell communication between keratinocytes and fibroblasts	
HELIAMI™ SEA FENNEL EC	Grows Mediterranean, Black Sea, Atlantic Ocean, and the Channel coast	It is rich in Vitamin C. The oily extract contains lipids, vitamins, and carotenes with regenerating and antioxidant properties	
SKIN TIGHTENER ST2™ association of a vegetable protein hydrolysate with polymannuronate	Macrocystis Pyrifera brown algae (Giant Kelp)	Provides an immediate skin-smoothing effect	Sederma by Croda Beauty (Snaith, UK)
ICHTYOCOLLAGEN™ PHSoluble collagen	Fish skin extract	Film forming and moisturizing	
